# The impact of a counselling intervention on nutrition practices among caregivers of children under two in the Kyrgyz Republic

**DOI:** 10.1017/S1368980024001307

**Published:** 2024-10-18

**Authors:** Aida Abdyldaeva, Nazgul Abazbekova, Aisuluu Abakirova, Tim Williams, Marie Paul Nisingizwe, Silvia Alayon, Jennifer Yourkavitch

**Affiliations:** 1 USAID Advancing Nutrition Kyrgyz Republic, Bishkek, Kyrgyz Republic; 2 John Snow, Inc., Arlington, VA, USA; 3 USAID Advancing Nutrition, Arlington, VA, USA; 4 University of British Columbia, Vancouver, Canada; 5 Save the Children, Washington, DC, USA; 6 Results for Development, Washington, DC, USA

**Keywords:** Nutrition behaviours, Nutrition programme impact, Social and behaviour change, Exclusive breastfeeding, Vitamin A-rich foods, Counselling

## Abstract

**Objective:**

To evaluate the impact of a counselling programme to strengthen the health and nutrition behaviours of caregivers of children under 2 and the sustainability of that impact through reduced intervention intensity one year later.

**Design:**

The programme trained community- and facility-based health staff to provide nutrition counselling. We conducted an impact evaluation with a modified stepped-wedge design using difference-in-differences analysis to compare indicator changes in an intervention group to a comparison group (midterm survey) and then a full intervention group to a light intervention group (final survey).

**Setting:**

Batken and Jalal-Abad oblasts, the Kyrgyz Republic, 2020–2023.

**Participants:**

Caregivers of children under 2 provided 6253 responses in three telephone surveys.

**Results:**

We observed statistically significant differences between the intervention and comparison groups at midterm for the percentage of children consuming vitamin A-rich foods; an increase in the intervention group (58–62 %) and a decrease in the comparison group (61–57 %). We observed similar results with exclusive breastfeeding (51–55 % in the intervention group and 48–40 % in the comparison group). There were also positive differences in other health and nutrition indicators. With the final survey results, in general, we observed statistically significant differences indicating a bigger change in full intervention areas compared to light intervention areas. We observed small negative changes in many indicators in light intervention areas.

**Conclusions:**

This evaluation highlights the importance of continued support for local interventions, particularly counselling programmes, to foster optimal nutrition behaviours.

Despite progress toward elimination, malnutrition among women and adolescent girls is still globally challenging^(1)^. Undernutrition (such as underweight and short stature), deficiencies in essential micronutrients and anaemia affect more than one billion adolescent girls and women globally, resulting in devastating consequences for their health and well-being^(1)^. According to a recent UNICEF report, progress may be slowing; between 2020 and 2022 there was a 25 % increase in acutely malnourished pregnant and breastfeeding women in 12 countries impacted by the current food and nutrition crisis^(1)^. Inadequate intake of nutrients before and during pregnancy and breastfeeding affects not only women but also their children^(2)^. Meanwhile, because of limited resources, the nutritional status of women of reproductive age is very poor^(3)^. Globally, an estimated two out of three women of reproductive age experience deficiency of at least one micronutrient^(4)^. For women, malnutrition during childhood may also affect birth outcomes, making them more likely to have difficult childbirths and lower birth weight infants^(3)^.

The Kyrgyz Republic has a population of about 6 million with high literacy and a wide coverage of health services, basic sanitation facilities and electricity^(5)^. However, an Integrated Context Analysis conducted by the World Food Programme shows that Batken and Jalal-Abad *Oblasts* (subregions) are the most vulnerable to poor nutrition because of high levels of poverty, food insecurity and vulnerability to natural disasters^(6)^. Of equal concern is the fact that a substantial proportion of the child population, especially children under 2 years, is malnourished. According to the most recent (2018) Multiple Indicator Cluster Survey in the Kyrgyz Republic, 12 % of children aged 0–5 years are stunted^(5)^. Jalal-Abad (16 %), Osh (14 %), Issyk Kul (14 %) and Batken (12 %) regions have the highest levels of childhood stunting^(5)^. At the same time, 7 % of children of the same age group were overweight^(5)^. This proportion reaches 9–12 % in some areas due to the high consumption of fatty and carbohydrate foods^(5)^. In the Kyrgyz Republic, the majority of nutrition services focused on the first 1000 days and are provided through the primary healthcare system during antenatal care visits and routine well-child visits. Although levels of access to health and nutrition services are high, the quality of those services remains poor^(7)^.

USAID Advancing Nutrition implemented a programme (‘the programme’) to improve nutrition for women and children under 2 years old in Batken and Jalal-Abad oblasts (Fig. 1). The programme’s objective was to sustainably improve the nutritional status of the Kyrgyz women and children, through the improvement of nutrition-related behaviours, strengthening the quality of nutrition services within the health system, and increasing the consumption of nutritious foods. Working in partnership with national and local governments, village health committees, oblast and district-level health centres and both local and international nongovernmental organisations, the programme promoted 11 evidence-based, nutrition practices (see online supplementary material, Supplementary Material 1) through community-based and facility-based counselling. Programme staff and activists promoted these practices using social and behaviour change approaches including improved health services and health worker capacity, community mobilisation and interpersonal communication at the community level and mass media in three districts in Batken and four districts in Jalal-Abad from 2020 to 2023.

**Fig. 1:**
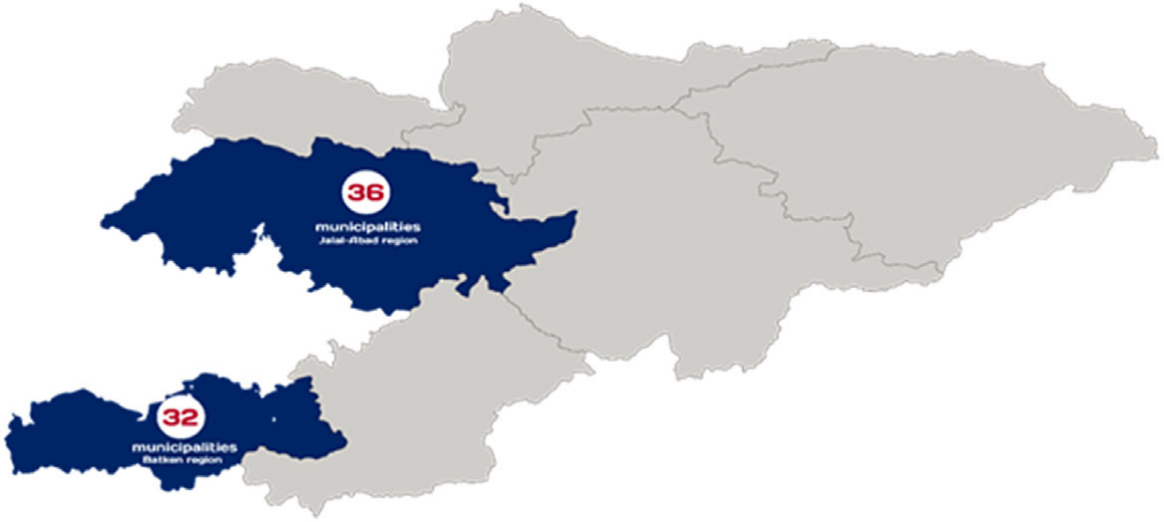
Programme implementation areas

Activists were volunteers who provided social and behaviour change support, mainly through household visits and community meetings, and ongoing contact through a chat message phone application. Programme staff recruited activists from the Kyrgyz Association of Village Health Committees. Kyrgyz Association of Village Health Committees is a non-profit association operating since 2010 with an aim of empowering and engaging rural people to address public health issues. Members of the Kyrgyz Association of Village Health Committees, operating in 1551 Village Health Committees across the country, cover 1431 villages in the Kyrgyz Republic, which is 81 % of all villages in the country. Community mobilisers, (programme staff), were hired locally in regions, according to the requirements in the job description and necessary to perform the job properly, including experience, knowledge and skills in working with communities.

Health facility-based support included updating national protocols and guidelines; training health workers; and integrating supportive supervision, mentorship and quality improvement approaches into routine care. The programme implemented a light intervention comprising less frequent household contacts by community activists with a streamlined refresher treatment of previous counselling topics during the year following the full intervention. At the national level, the programme advocated for improved policies and resource allocation for nutrition services and worked to strengthen local implementing partners, such as the Kyrgyz Association of Village Health Committees. This paper presents an evaluation of the programme’s impact on health and nutrition behaviours of caregivers of children under 2 and the sustainability of that impact through reduced intervention intensity one year later.

## Methods

### Study design

For this impact evaluation, we used a modified stepped-wedge design to examine the effect of programme interventions on 20 indicators associated with the 11 evidence-based, nutrition-related practices. Our programme design ensured that everyone in the programme area could participate and our evaluation design enabled us to measure effects. We compared indicators before and after the intervention in the intervention and comparison areas and then examined the sustainability of the outcomes after one year of full intervention followed by one year of light intervention. Due to limited travel and close contact from the COVID-19 pandemic, we hired a firm to conduct the baseline, midterm and endline surveys and to analyse the data. The firm conducted telephone interviews with cross-sections of the target population using computer-assisted telephone interview technology, in the mid-to-late fall of 2020, 2021 and 2022.

Prior to the baseline survey, the study administrative team (managers and evaluation experts) randomly assigned villages within Batken and Jalal-Abad Oblasts to either intervention or comparison groups. Intervention areas benefited from a full range of programme activities (including community- and facility-based counselling). Community-based counselling was conducted virtually due to COVID-19 restrictions on in-person contact. These contacts involved counselling with modules on different maternal and child health and nutrition themes. Comparison groups were exposed only to mass media. After one year of implementation, we carried out a midterm survey and compared difference-in-differences (DiD) estimates between intervention and comparison areas to assess programme impact. Baseline and midterm surveys also highlighted those practices where prevalence of healthy behaviours was low and where programme interventions could effectively focus during the remainder of the programme. After the midterm survey, villages in the original comparison areas began receiving full programme interventions for the second year of the study (Table 1; the stepped wedge). Villages in the original intervention areas continued receiving programme support, but at a substantially reduced level compared to what they received in the first year of the study (light intervention). There were 6–8 household contacts during the full intervention and 4 household contacts during the light intervention, with some of the latter in person as pandemic restrictions were eased. Examining indicators in the light intervention group at the final survey point enables us to ascertain the near-term sustainability of the intervention’s effects with a substantially reduced intervention effort. The timing of the programme interventions and three surveys is shown in Table 1.

**Table 1:**
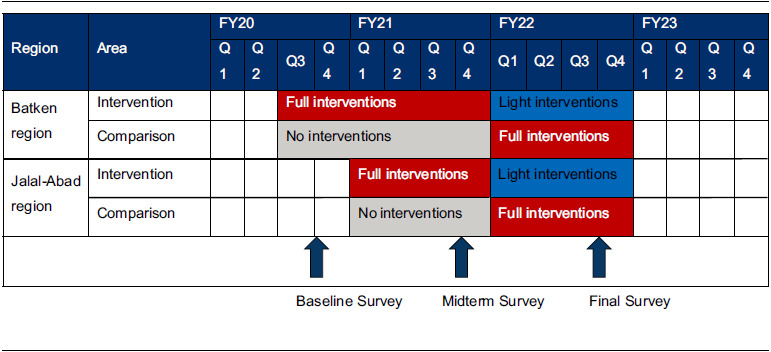
Timing of programme interventions and assessments

### Study population

The survey interviewed women aged 18 years and older, with at least one child aged 0–23 months, living in the intervention and comparison villages of Jalal-Abad and Batken regions (Fig. 1). Enumerators carried out the survey in 36 *Ayil Aimaks* (municipalities) in Jalal-Abad region and 32 Ayil Aimaks in Batken region, which included 227 settlements in Jalal-Abad and 144 settlements in Batken.

We identified respondents through health facility records. Virtually, all women with children register with a nearby health facility, and facilities keep records of those clients, including age of the child and address and phone number of the mother. We obtained lists of phone numbers from all health facilities in both intervention and comparison areas. The computer-assisted telephone interview programme randomly chose phone numbers from these lists, until the desired sample size was reached for a given respondent type (e.g. child 0–5 months, child 6–23 months, in intervention and comparison areas in each oblast). Ultimately, because of high levels of non-response (typical for phone surveys) enumerators called all numbers on the lists and then supplemented with phone numbers from other databases (e.g. lists from baseline and midterm surveys).

### Sampling and sample size

To estimate a total required sample size, we determined a minimum sample size for surveys to measure changes in key indicators for two child age groups (0–5 months and 6–23 months) within two treatment classifications (intervention and comparison) in the two regions (see online supplementary material, Supplemental Material 2). The desired sample size was 385 for each sub-category, for a total desired sample size of 3080. The ultimate sample sizes were 2091 for the baseline, 2234 for the midterm and 1928 for the endline.

The shortfall was primarily due to high levels of non-response, outdated lists of numbers from health facilities and non-functional phone numbers. The main reasons for the changes in sample size across the three surveys were variation in the number of phone numbers provided by health facilities in the survey areas and the higher percentage of non-functional phone numbers on the endline survey lists. The military conflict on the border between Batken and Tajikistan caused substantial displacement of households in Batken and further reduced the final survey sample size.

If there was more than one child under two years in a household, enumerators asked questions about only one child. In such cases, if one of the children was 0–5 months old, that child was selected until we achieved the desired number of children 0–5 months of age. With fewer children in that age group than the 6–23-month age group, prioritising them improved our chance of achieving the desired number of interviews associated with the youngest children. After we achieved the desired sample of children 0–5 months, if a selected household had two or more children under two years of age, enumerators asked the mother questions about the child that had the last (most recent) birthday.

### Survey questionnaire

The survey questionnaire contained 12 modules, assessing women’s diets and children’s nutrition, among other health areas, including breastfeeding practices, meal frequency, dietary diversity and overall diet quality (see online supplementary material, Supplementary Material 3). We pre-tested the questionnaire before the baseline and midterm surveys and modified where needed based on that experience. In the baseline survey, the full questionnaire took 31 min on average. Experience with phone surveys at the time suggested that amount of time was slightly longer than the recommended interview times, and respondents would refuse at the beginning or drop off during the call if it lasted longer^(8)^. We quickly achieved the desired sample size for indicators that were asked of all respondents, regardless of the child’s age (e.g. indicators related to iron consumption, handwashing, and food storage, among others). It was also relatively easy to achieve the desired sample size for indicators asked only of children 6-23 months (e.g. children’s dietary diversity). For indicators that were asked only for children 0–5 months, however (e.g. exclusive breastfeeding (EBF)), we had difficulty obtaining the desired number of interviews. Therefore, we used a crude form of block randomisation, in which enumerators would ask respondents all questions appropriate to the age of their child until the desired sample size was reached for indicators related to children aged 6-23 months. After that, we stopped interviewing mothers of children aged 6-23 months and asked women with a child 0-5 months only those modules related to that age (see online supplementary material, Supplementary Material 2). Those women were asked questions from a much shorter questionnaire focused on indicators associated with that age group. The short interview took only 13 min to complete. Respondents who fully completed their interview received a small credit to their phone account (50 KGS = 0·60 USD or about 30–45 min).

Before the midterm survey, we added a module on gender and household decision-making and more detailed questions about respondents’ exposure to programme activities and messages. This made interviews with the full questionnaire over 40 min. To keep interviews at a reasonable length for phone interviews, we again used a modified form of block randomisation or parallel sampling described in online supplementary material, Supplementary Material 2.

### Data analysis

We used DiD analysis with regression equations using IBM SPSS Statistics for Windows, version 29 (IBM Corp., Armonk, NY, USA) to compare changes in the intervention *v*. comparison areas between the baseline and midterm surveys. For the final survey, we used the same DiD approach, this time comparing changes in the full intervention areas to changes in the light intervention areas. The null hypothesis for the DiD analyses was that in the absence of (or light intervention) programme activities, the difference between the areas would be constant over time. Analysis for each indicator was done separately for Batken and Jalal-Abad oblasts and for the full dataset with both oblasts. We tested the statistical significance (*P* < 0·05) of each DiD estimate using a simple regression model, with DiD expressed as an interaction term between time and a treatment group dummy variable. We tested demographic differences between intervention and comparison groups at each timepoint using R (version 4·3·1).

## Results

### Description of the surveyed population

Our response rate among the eligible women we reached ranged from 41 % at baseline to 26 % at midterm and 18 % at endline. Populations in the intervention and comparison groups were comparable at all survey time points except for maternal age in the final survey, which was slightly younger in the light intervention area compared to the full intervention area (Table 2). The average age of women at all time points was 29. One-third of women have at least general secondary education (11 years of school) and most were married in each group at each survey timepoint.

**Table 2:** Respondent demographics (percentages)

Characteristic	Baseline			Midterm			Final		
	Intervention (*n* 1125)	Comparison (*n* 966)	*P*-value	Intervention (*n* 1132)	Comparison (*n* 1102)	*P*-value	Light intervention (*n* 955)	Full intervention (*n* 973)	*P*-value
Mother age									
18–24 years	26·3	25·0	0·96	22·6	21·8	0·46	22·2	24·3	0·04*
25–29 years	34·8	35·2		34·8	36·4		36·9	32·3	
30–39 years	35·7	36·7		40·4	38·8		38·3	39·1	
40–49 years	3·1	3·2		2·2	3·1		2·5	4·2	
50+ years	0·0	0·0		0·0	0·0		0·1	0·0	
Education									
None	0·4	0·0	0·20	0·2	0·1	0·14	0·0	0·1	0·94
Primary	0·2	0·1		0·4	0·1		0·1	0·1	
Basic secondary (9 grades)	6·0	5·3		8·9	9·0		8·9	8·0	
General secondary (11 grades)	38·5	38·1		32·5	31·2		31·1	33·0	
Initial vocational education	3·7	2·9		3·1	1·6		2·1	2·2	
Secondary specialised education	24·9	23·4		25·6	25·9		26·0	26·0	
Higher education (incomplete or completed)	26·3	30·2		29·2	31·7		31·8	30·6	
Marriage/partnership									
Married	98·6	97·9	0·63	97·8	98·8	0·13	97·8	98·4	0·56
Divorced	0·5	0·8		1·3	0·5		1·4	1·0	
Single	0·7	0·8		0·7	0·6		0·6	0·6	
Widow	0·2	0·4		0·2	0·1		0·2	0·0	
Child age									
0–5 months	29·2	22·4	<0·01	31·0	29·0	0·58	35·6	35·1	0·20
6–11 months	25·6	27·1		27·3	27·0		27·3	37·7	
12–17 months	24·8	31·6		23·7	24·1		23·7	15·0	
18–23 months	20·4	18·9		18·0	20·0		18·0	12·2	
Child sex									
Male	52·5	51·4	0·59	51·2	51·0	0·94	51·5	48·8	0·24
Female	47·8	48·7		48·9	49·0		48·9	51·2	
Jalal-Abad	33·5	48·9		46·5	54·5		38·4	60·8	

* Indicates a statistically significant difference (*P* < 0.05) between intervention and comparison group.

### Main results

**Fig. 2:**
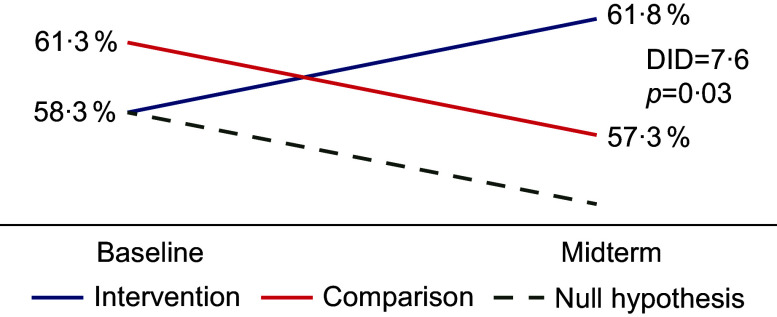
Impact of intervention on children’s consumption of vitamin A-rich foods

**Fig. 3:**
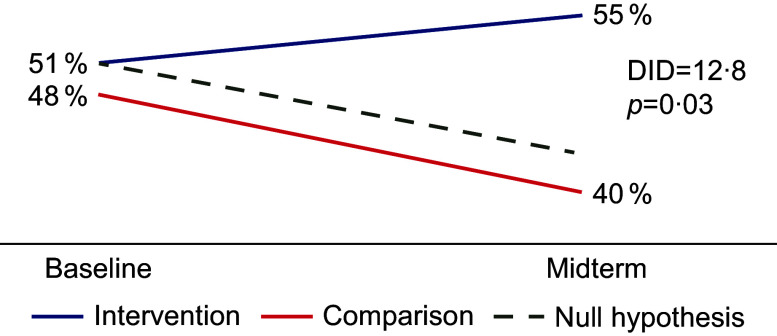
Impact of intervention on exclusive breastfeeding for children under 6 months of age

We observed statistically significant difference estimates for two indicators at midterm: children consuming vitamin A-rich foods (Fig. 2) and exclusive breastfeeding (Fig. 3), although there were positive differences in several other indicators, indicating a bigger positive change in intervention areas than in comparison areas (Table 3) and suggesting the impact of the programme’s efforts. With the final survey results, we observed more statistically significant differences, indicating a bigger change in intervention areas compared to light intervention areas. We observed decreases in many indicators in the areas that transitioned from full intervention to light intervention, but most decreases were small. Charts for each indicator are shown in online supplementary material, Supplementary Material 4, and a table of changes by region is included in online supplementary material, Supplementary Material 5.

**Table 3:** Indicator changes, both regions

Indicator	Baseline				Midterm				Difference (intervention [M-B] – comparison [M-B])*	Final				Difference (full intervention [F-M] – light intervention [F-M])*
	Intervention		Comparison		Intervention		Comparison			Light intervention		Full intervention		
	*n*	%	*n*	%	*n*	%	*n*	%		*n*	%	*n*	%	
Percent of mothers of children <2 who took iron supplements for 90 d or more during their last pregnancy†	394	47·9	372	46·3	486	58	483	56·8	−0·4	423	59·2	399	54·8	−3·2
Percent of mothers of children <2 who ate foods from 5 or more of 10 food groups in the previous 24 h^(Adapted from(2)^	738	90·4	675	85·8	738	88·2	748	88	−4·4	612	85·8	621	85·5	−0·1
Minimum dietary diversity: percent of children 6–23 months who ate foods from 5 or more of 8 food groups in the previous 24 h^(9)^	512	64·8	491	66·5	505	64·7	494	63·2	3·4	353	57·4	357	56·6	0·8
Minimum acceptable diet: percent of children 6–23 months receiving a minimum acceptable diet^(9)^	129	17·7	115	16·3	136	18·8	106	14·8	2·6	94	16·4	114	19·8	7·4*
Percent of children 6–23 months who ate iron-rich foods in the previous 24 h^(9)^	499	62·7	486	64·8	516	66·2	492	62·9	5·3	356	57·9	366	57·9	3·3
Percent of children 6–23 months who ate vitamin A-rich foods in the previous 24 h^(9)^	464	58·3	460	61·3	482	61·8	448	57·3	7·6*	339	55·1	338	53·5	2·9
Minimum meal frequency: percent of children 6–23 months who received food the minimum acceptable number of times for their age and breastfeeding status^(9)^	177	24·5	163	23·5	187	25·8	147	20·5	4·1	155	27·0	167	29·0	7·3*
Early initiation of breastfeeding: Percentage of children 0–23 months who were put to breast within one hour of birth^(9)^	709	63·3	602	62·4	799	70·8	459	69·1	0·8	644	67·7	651	67	1
Exclusive breastfeeding: percentage of infants 0–5 months of age who were fed exclusively with breast milk during the previous day^(9)^.	168	51·1	104	48·2	195	55·4	127	39·7	12·8*	186	54·7	211	61·8	22·9*
Percent of children 6–8 months who received semi-solid or solid food during the previous 24 h (without sweet, processed products^)(Adapted from^ ^(9)^	119	88·2	111	86·1	141	84·4	137	81. 1	1·3	137	82	168	81·6	2·9
Percent of children 6–23 months who are still breastfeeding^(Adapted from^ ^(9)^	644	81·3	626	83·5	650	83·7	644	82·6	3·3	522	85·4	542	86	1·7
Percent of children 0–5 months who consumed sugary or processed food in the previous 24 h^(Adapted from^ ^(9)^	49	14·9	31	14·4	41	11·7	57	17·8	−6·7	39	11·5	32	9·4	−8·3*
Percent of children 6–23 months who consumed sugary or processed food in the previous 24 h^(Adapted from^ ^(9)^	678	85·2	649	86·5	611	78·3	621	79·4	0·3	440	71·5	423	66·9	−5·7
Percent of children 0–5 months who consumed tea in the previous 24 h^(Adapted from^ ^(9)^	41	12·5	30	13·9	30	8·5	44	13·8	−3·8	32	9·4	25	7·3	−7·3*
Percent of children 6–23 months who consumed tea in the previous 24 h^(Adapted from^ ^(9)^	584	73·4	545	72·7	502	64·5	532	68	−4·4	354	57·6	338	53·5	−7·8*
Percent of women who received advice to take deworming medicine during pregnancy	168	20·4	155	19·3	165	19·6	121	14·7	3·7	142	19·4	120	16	1·5
Percent of women who usually wash hands at least three out of five critical times‡	295	35·9	245	30·5	336	39·7	259	31·5	2·9	236	32	231	30·8	7·0*
Percent of households with soap and water at a handwashing station on premises‡	818	99·5	799	99·5	844	99·7	817	99·8	0·5	733	99·5	744	99·2	0·2
Percent of women who stored or preserved nutrient-dense products for consumption during the last winter	774	94·2	762	94·9	823	94·2	781	94·1	0·9	638	89·2	667	90·5	1·3
Percent of women reporting increased decision-making power with husband and/or family	Not collected		Not collected		255	96·2	265	98·9	N/A	305	98·4	307	97·8	−3·3

* Statistically significant at *P* < 0.05.

† Adapted from Demographic Health Surveys (Croft, Trevor N., Allen, Courtney K., Zachary, Blake W., *et al.* 2023. Guide to DHS Statistics. Rockville, Maryland, USA: ICF).

‡ World Bank. 2019. ‘Nutrition-Sensitive Water Supply, Sanitation, and Hygiene’. World Bank, Washington, DC.

#### Infant and young child feeding

The percentage of children that were put to breast within the first hour of birth increased among the intervention group before decreasing slightly after the light intervention (63 % at baseline, 71 % at midline, 68 % at endline; Table 3). Over time, the pattern was similar for the comparison group, decreasing slightly after they received the full intervention (62 % at baseline, 69 % at midline, 67 % at endline). The percentage of children 0–5 months of age who were exclusively breastfeeding increased for the intervention group and held steady during their light intervention phase (51 % at baseline, 55 % at midline, 55 % at endline). Over the same period, that percentage decreased among the comparison group, before substantially increasing after their full intervention phase (48 % at baseline, 40 % at midline, 62 % at endline; Table 2). More than 80 % of mothers of children aged 6–23 months in both groups and regions were breastfeeding at each survey point. The final survey showed that the most common reasons for stopping breastfeeding were pregnancy (31 %), mother’s illness or treatment with antibiotics (13 %), a lot of or too little milk (11 %) and the mother’s need to return to work (12 %). On average, the duration of breastfeeding was 14 months.

We observed a steady decline in consumption of semi-solid or solid foods among children aged 6–8 months in the first intervention area and through the light intervention phase (88 % at baseline, 84 % at midline, 82 % at endline); the comparison area held steady through its intervention phase (86 % at baseline, 81 % at midline, 82 % at endline; Table 3). We observed a steady decrease in the percentage of children under 2 consuming sugary or processed foods such as chocolate, sweets, pastries, cakes or cookies in the intervention group, maintained through the light intervention phase (15 % at baseline, 12 % at midline, 12 % at endline). At the same time, consumption among the comparison group increased (14 %–18 %) before decreasing during their full intervention phase (9 %; Table 3).

During the same time, there was a reduction in the consumption of various types of cereals, *bylamyk* (porridge), etc., other fruits and vegetables and root vegetables. The most commonly consumed food groups in every survey were grains (e.g. bread, rice, buckwheat, maize, noodles or other cereals) and other fruits and vegetables, including apple, banana, dates, grapes, kiwi, lemon, tangerine, orange, pear, pineapple, plum, pomegranate (anar), cherry, raspberry, strawberry and watermelon. The least consumed food groups among those mentioned by respondents in all three surveys were flesh foods (fish and seafood, liver, kidney, heart, stomach or other organ meats) and dark green leafy vegetables such as broccoli, spinach and sorrel. Consumption of tea among children aged 6–23 months decreased steadily in the initial intervention group (73 % at baseline, 65 % at midline, 58 % at endline) and the initial comparison group (73 % at baseline, 68 % at midline, 53 % at endline; Table 3).

The minimum dietary diversity (MDD) of children aged 6–23 months is achieved if the child consumes foods from at least five of eight food groups^(9)^. Among children aged 6–23 months in both groups, 65 % consumed at least 5 or more food groups at baseline and midterm (Table 3). This result dropped to 57 % during the light intervention phase. In the comparison group, this dropped from 67 % to 63 % and then further dropped to 57 % during its full intervention phase. In the intervention group, only 24 % of children aged 6–23 months received the minimum number of meals (MMF: minimum meal frequency) for their ages at baseline. This percentage steadily increased to 26 % and then 27 % during the light intervention phase. This percentage decreased in the comparison group from 23 % at baseline to 20 % at midline but then increased to 29 % during the full intervention phase (Table 3).

#### Micronutrients

In the intervention group, the percentage of children aged 6–23 months consuming iron-rich foods increased from 63 % to 66 % but then decreased to 58 % during the light intervention phase. Meanwhile, that percentage steadily decreased in the comparison group, even after their full intervention phase (65 % at baseline, 63 % at midline and 58 % at endline; Table 3). The percentage of children aged 6–23 months consuming vitamin A-rich foods increased in the intervention group before decreasing after the light intervention phase (58 % at baseline, 62 % at midline and 55 % at endline; Table 3). There was a steady decrease in the comparison group, even during the full intervention phase (61 % at baseline, 57 % at midline and 54 % at endline; Table 3). The percentage of mothers of children under 2 who took iron supplements for at least 90 days during their last pregnancy steadily increased in the intervention group (48 % at baseline, 58 % at midline and 60 % at endline) and generally increased among the comparison group (46 % at baseline, 57 % at midline and 55 % at endline; Table 3).

#### Other indicators for women and households

The percentage of women washing their hands at recommended times increased in the intervention group from 36 % to 40 % and then decreased during the light intervention phase to 32 % (Table 3). Meanwhile, that percentage among the comparison group did not change (31 % at baseline, 32 % at midline and 31 % at endline). Other indicators such as the percent of households with soap and water for handwashing and the percent of women with increased decision-making power were high at baseline and remained so throughout survey points. The percent of women who stored or preserved nutrient-dense foods for consumption during winter remained high but decreased slightly for both groups at the time of the final survey (Table 3). Finally, the percentage of women who received advice about taking deworming medicine during pregnancy held steady for the intervention group (20 % at baseline, 20 % at midline and 19 % at endline) but was more variable for the comparison group, decreasing first and then increasing slightly when they received the full intervention (19 %–15 %–16 %; Table 3). The percentage of women eating the recommended number of food groups decreased steadily in the intervention group (91 % at baseline, 88 % at midline and 86 % at endline), while it remained the same for the comparison group even after they received the full intervention (86 % at baseline, 88 % at midline and 86 % at endline; Table 3).

## Discussion

We conducted an impact evaluation to measure a programme’s effects on health and nutrition behaviours and the sustainability of those effects through reduced programme intensity one year later. We used a modified stepped-wedge evaluation design to enable our examination of the programme’s impact and near-term sustainability and to ensure equity in terms of the whole programme area population having the chance to receive the interventions. We found the greatest programme impact on EBF among children under 6 months of age and on consumption of vitamin A-rich foods among children aged 6–23 months. We also found that the percentage of the population with optimal breastfeeding behaviours held steady through the light intervention phase. The percentage of children under 2 consuming sugary and processed foods decreased during the intervention, which is a positive result, but, in general, other indicators measuring positive aspects of children’s diets also steadily declined for both groups, possibly reflecting environmental shifts and lack of food availability during the COVID-19 pandemic.

The WHO recommends EBF for children under the age of 6 months, and beginning at six months of age, children should consume soft and semi-solid foods while continuing to receive breast milk through at least the age of 2 years^(9,10)^. Nearly all mothers in the Kyrgyz Republic report breastfeeding their children; however, according to the 2018 Multiple Indicator Cluster Survey, only 46 % of children between 0 and 5 months are exclusively breastfed^(5)^. We found a slightly higher prevalence in the programme area (51 %) and observed an increase during the programme (55 %). That increase was four percentage points in the first phase, measured after the intensive intervention and that increase held steady through the ‘light intervention’ phase in that area, keeping the prevalence above the World Health Assembly target of 50 % by 2025^(11)^. We observed a substantial increase among the comparison group who received the intensive intervention in the second phase (about 22 percentage points), bringing that area closer to the other in terms of EBF prevalence and surpassing the World Health Assembly target. The second area (comparison) group had a lower starting prevalence so there was more to gain and counsellors were more experienced by the time that area received the intensive intervention which may account for the larger increase in that area.

According to the 2018 Multiple Indicator Cluster Survey, 60 % of children under the age of 2 receive the recommended number of food groups throughout the country, three-quarters receive the number of recommended feedings for their age, and 43 % receive a minimum acceptable diet^(5)^. In the programme area, we found a similar percentage receiving the recommended number of food groups but a drastically lower percentage receiving the appropriate number of feedings (around 25 %) which may have been due to the recent changes in conditions (pandemic and conflict).

We found a few other comparable studies and they had similar results. In Burkina Faso, government researchers trained facility-based health care providers in nutrition counselling and then followed a cohort of pregnant women until their children were 18 months old^(12)^. They found that mothers who received the intervention were more likely to EBF and their children were more likely to have dietary diversity^(12)^. An impact evaluation of an interpersonal counselling intervention to improve children’s nutrition in Bangladesh found slight declines in infant and young child feeding indicators two years after the intervention ended, but sustained improvement over baseline measures^(13)^. Another trial in Bangladesh tested digital job aids for community health workers (along with lipid-based supplements and concluded that nutrition counselling provided by community health workers with a digital job aid improved children’s dietary diversity^(14)^.

As the world emerges from the COVID-19 pandemic, it remains to be seen how nutrition programmes fared. In general, global food scarcity has increased in the past few years and estimates of maternal and child malnutrition have increased^(15)^. Nutrition programmes focused on attainable behaviour goals such as changing breastfeeding behaviours were likely more successful than those with goals that could be influenced by external forces including economic and environmental shifts on access to food. In contrast, handwashing is affected by many factors, including water source and availability, weather, type of employment, etc., some of which are beyond the scope of health or nutrition projects to influence. We observed an increase in handwashing during the intervention followed by a decrease during the light intervention; past projects in Kyrgyz Republic have also experienced challenges in improving handwashing indicators, especially during cold weather months^(16)^.

According to WHO, children are especially susceptible to iron deficiency anaemia due to the increased need for iron during periods of rapid growth, mainly in the first five years of life^(17)^. Iron deficiency anaemia in children is associated with increased levels of morbidity, as well as impaired cognitive development and poor school performance^(18)^. Iron is found both in animal products (meat, by-products, poultry and fish) and produce (legumes, spinach, apples, cereals, nuts and dried fruits). In 2021, the prevalence of iron deficiency anaemia among children 6-59 months was estimated at 18 % in Batken and 6 % in Jalal-Abad^(19)^. Overall, we found a decrease in children’s consumption of iron-rich foods over time in both intervention and comparison groups, especially between midterm and endline surveys, which may reflect the effects of economic and environmental shifts on food availability during the COVID-19 pandemic.

Consumption of vitamin A-rich foods is important because vitamin A promotes rapid growth in infants and young children and helps them fight infections^(20)^. Insufficient intake of vitamin A can lead to visual impairment in the form of night blindness and increase the risk of morbidity and mortality from childhood infections, including measles and intestinal infection^(20)^. The prevalence of vitamin A deficiency in 2021 was 18 % in Batken and 12 % in Jalal-Abad^(19)^. The programme demonstrated an impact on the consumption of vitamin A-rich foods; the percentage of children consuming in the intervention group increased (four percentage points) while that percentage decreased in the comparison group. Thus, the effects of interpersonal counselling may have protected against a decrease in the general population. However, we observed that this effect disappeared after the full intervention phase, again pointing to the importance of food availability (not addressed by the programme) in addition to a person’s ability to change their behaviour. In addition, the increases and decreases were slight so they may not be programmatically meaningful. However, it is difficult to draw a conclusion about that from just one year of implementation. Similarly, the observed drop in MDD during the full intervention phase in the comparison group and the low MMF overall may indicate a lack of food availability as well as customs and beliefs, e.g. mothers-in-law and husband typically making decisions about purchasing, planning, and preparing food, with a preference for traditional dishes, and inflexibility around meal times due to work schedules.

There are limitations to this evaluation. The surveys occurred just one year after each other and the programme implementation schedule was such that some areas did not receive the full interventions beforehand. While it is important to measure the impact of health and nutrition programmes, measurement should occur at time points that allow for adequate programme implementation. One year of implementation is likely not enough to see substantial changes in most population health and nutrition indicators. In addition, interviews were conducted by phone due to the pandemic and that contributed to a low response rate (41 % at baseline, 26 % at midterm and 18 % at endline), which may have biased the estimates in an undetermined way if women with certain characteristics were more likely to complete the surveys while others were not. However, our response rates were not very different from other studies using phone surveys in low- and middle-income countries^(21,22)^. Most people (93 %) have access to a cell phone^(23)^. We conducted three cross-sectional surveys, which limits trend analysis for or examination of individual behaviours by examining population behaviours in aggregate over time. There was in- and out-migration from the programme area, especially out-migration from Batken during the conflict with Tajikistan, and that likely changed the surveyed population in terms of the number of health service contacts that could be experienced by respondents at different time points, in addition to changing the environment in which those respondents lived. There may have been spillover due to caregivers from comparison areas accessing care from facilities in intervention areas which may have attenuated the observed effects. Finally, responses were subject to possible recall bias and social desirability bias.

Nonetheless, this programme and evaluation offer important lessons for programmes focused on increasing capacities for community- and facility-based nutrition counselling. During a time of conflict or increasing food scarcity, and especially when those coincide, the health behaviours that can change sustainably are those that can persist regardless of shifting economic and environmental factors, such as breastfeeding. When circumstances permit, such as a global pandemic or even severe weather that keeps people indoors, nutrition programmes can use the opportunity to promote optimal breastfeeding practices. We found that population-level improvements in those behaviours were particularly sustainable through crises and over time. Future research could examine the sustainability of interventions over a longer period of time. This evaluation contributes to the growing body of evidence on the role of counselling interventions such as the programme evaluated here in fostering better nutrition behaviours such as EBF and consumption of vitamin A-rich foods, highlighting the importance of continued support for such interventions.

## Supporting information

Abdyldaeva et al. supplementary material Abdyldaeva et al. supplementary material

## Supplementary material

For supplementary material accompanying this paper visit https://doi.org/10.1017/S1368980024001307
