# Supplementary material for: The impact of a counselling intervention on nutrition practices among caregivers of children under two in the Kyrgyz Republic
Source: Public Health Nutr. 2024 Oct 18;27(1):e214. doi: 10.1017/S1368980024001307 (PMC11604319; doi:10.1017/S1368980024001307)
Supplement: Abdyldaeva et al. supplementary material [file S1368980024001307sup001.docx]

**Supplementary Material 1: Evidence-based, nutrition practices promoted by the program***

1. Consumption of iron-folic acid (IFA) supplements by pregnant women
2. Dietary diversity for women, with an emphasis on consumption of food sources of iron and foods that enhance iron absorption
3. Dietary diversity for children 6–23 months, with an emphasis on consumption of food sources of iron and vitamin A, and foods that enhance iron absorption
4. Minimum meal frequency for children 6–23 months of age
5. Early initiation of breastfeeding
6. Exclusive breastfeeding from birth through the first 6 months
7. Timely introduction of appropriate complementary foods
8. Reduced consumption of high-calorie, low-nutrient-density (junk) food
9. Presumptive treatment of helminth infections for pregnant women and children
10. Handwashing at five critical times: after using the latrine, after changing a baby’s diaper/cleaning a child, after handling animals, before preparing food, and before feeding a child
11. Adoption of methods for safe and prolonged storage of nutrient-dense produce for the winter.

*For more information, see: [USAID Advancing Nutrition Kyrgyz Republic: Strengthening National Efforts to Improve Nutrition | USAID Advancing Nutrition](https://www.advancingnutrition.org/resources/usaid-advancing-nutrition-kyrgyz-republic-strengthening-national-efforts-improve)

**Supplementary Material 2: Sampling information**

The target population for this survey will be women with children under the age of two, in Batken and Jalal-Abad Regions. We developed a questionnaire to measure 21 outcome indicators of interest associated with the 11 nutrition-related practices that the project is trying to improve. Some indicators were measured among women with children 0–23 months old, some among those with children 0–5 months, and some among women with children 6–23 months. To estimate a total required sample size, we determined a minimal sample size for pre- and post-surveys for each group of interest, to measure changes in exclusive breastfeeding (among children 0–5 months) and minimum acceptable diet (among children 6–23 months. The groups of interest were as follows:

● Batken intervention—children 0–5 months

● Batken intervention—children 6–23 months

● Batken comparison—children 0–5 months

● Batken comparison—children 6–23 months

● Jalal-Abad intervention—children 0–5 months

● Jalal-Abad intervention—children 6–23 months

● Jalal-Abad comparison—children 0–5 months

● Jalal-Abad comparison—children 6–23 months

We used the following equation to calculate the desired sample for each of the above subgroups:


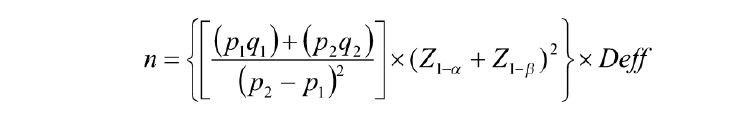


Where *p*_1_ and *p*_2_ are the values of the key indicators at times 1 and 2 respectively, *q*_1_=1-*p*_1_, *q*_2_=1-*p*_2_, a is the Type 1 error, (1-*B*) is the power and Z_1_-*a* and Z_1_-*B* are the standard Z-scores at the set levels of *a* and *B*, and Deff is the design effect from a previous comparable survey. The sample size is estimated based on a confidence level of 95%, a power of 0.8, and detecting a change of 10 percentage points between surveys, with a design effect = 1.0.

Based on those parameters, we determine a desired sample size of 385 for each of the above eight subgroups, for a total desired sample of 3,080 completed interviews. The calculation was as follows:

**
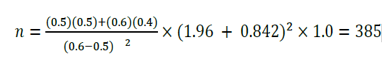
**

**Mid-term survey block randomization approach**

In this approach, enumerators asked all respondents four questionnaire modules, constituting one “block” of questions, while 4 other blocks of questions were developed, each with 6 out the 8 non-mandatory modules (see table below). After completing the four mandatory modules, each respondent would be randomly selected for one of 4 blocks, in such a way that each module would be answered by approximately 75% of respondents. The following table shows the distribution of modules in each block for the midterm and endline surveys, with numbers of respondents in the endline in each block and module.

*Table 2. Distribution of the questionnaire by modules according to the block randomization, fin. N=1928*

| **Block number** | **Block composition** | **Decryption of modules** |
| --- | --- | --- |
| **Mandatory block for all, n =1,928** | Modules A, C, F, K1 | MODULE A: Introduction and Informed Consent, n=1,928  MODULE C: Household Roster, Socio-economic and Demographic Data, n=1,928  MODULE F: Children’s Nutrition, n=1,928  MODULE K1: USAID Advancing Nutrition Exposure Questions, n=1,928  MODULE D: Maternal Nutrition and Antenatal Care, n=1,442  MODULE E: Women’s Dietary Diversity, n=1,442  MODULE G: Deworming, n=1,487  MODULE H: Water, Sanitation, and Hygiene, n=1,487  MODULE I: Food Storage and Preservation, n=1,456  MODULE J: Television and Social Media, n=1,456  MODULE K2: Knowledge questions, n=1,399  MODULE L: Gender and Decision Making, n=1,399 |
| **Block 1, n=529** | Modules D, E, G, H, I, J |  |
| **Block 2, n=486** | Modules G, H, I, J, K2, L |  |
| **Block 3, n=472** | Modules D, E, G, H, K2, L |  |
| **Block 4, n=441** | Modules D, E, I, J, K2, L |  |

This approach allowed us to maximize our potential sample size for each indicator, while keeping interview times similar to the baseline survey even with the addition of a new module and additional questions about program exposure. The total interview duration averaged 32 minutes, with insignificant time differences between the four blocks.

The achieved sample size in the three surveys is shown in the table below:

|  | **Region** | **Intervention/Comparison** | **Child age** | **N of respondents** | | |
| --- | --- | --- | --- | --- | --- | --- |
|  |  |  |  | Baseline (BL): 2022 | Midterm (MT): 2023 | FN: 2024 |
|  | Jalal-Abad region | Intervention zone (BL to MT)  Light Intervention  (MT to EL) | 0–5 months | 73 | 135 | 128 |
|  |  | Intervention zone (BL to MT)  Light Intervention  (MT to EL) | 6–23 months | 304 | 391 | 238 |
|  |  | Comparison zone  (BL to MT)  Intervention zone  (MT-EL) | 0–5 months | 102 | 212 | 230 |
|  |  | Comparison zone  (BL to MT)  Intervention zone  (MT-EL) | 6–23 months | 371 | 388 | 362 |
|  |  | ***Total on Jalal-Abad intervention/Light Intervention zone*** | | 377 | 526 | 366 |
|  |  | ***Total on Jalal-Abad Comparison/Intervention zone*** | | 473 | 600 | 592 |
|  | **TOTAL FOR JALAL-ABAD REGION** | | | **850** | **1126** | **958** |
|  | Batken region | Intervention zone (BL to MT)  Light Intervention  (MT to EL) | 0–5 months | 256 | 217 | 212 |
|  |  | Intervention zone (BL to MT)  Light Intervention  (MT to EL) | 6–23 months | 492 | 389 | 377 |
|  |  | Comparison zone  (BL to MT)  Intervention zone  (MT-EL) | 0–5 months | 114 | 108 | 111 |
|  |  | Comparison zone  (BL to MT)  Intervention zone  (MT-EL) | 6–23 months | 379 | 394 | 270 |
|  |  | ***Total on Batken Intervention/Light Intervention zone*** | | 748 | 606 | 589 |
|  |  | ***Total on Batken Comparison/ Intervention zone*** | | 493 | 502 | 381 |
|  | **TOTAL FOR BATKEN REGION** | | | **1241** | **1108** | **970** |
|  | **Total** |  |  | **2091** | **2234** | **1928** |

**Supplementary Material 3: Survey Instrument**

| **Modules** | **Instructions and Indicators** |
| --- | --- |
| **Module A:** Introduction and Informed Consent | All women contacted must give consent to be surveyed |
| **Module B:** Household Identification Cover Sheet | To be filled by enumerator [auto-filled section before or after interview] |
| **Module C:** Household Roster, Socio-economic and Demographic Data | Only women above 18 should be interviewed starting with this module |
| **Module F:** Children’s Nutrition | **(Practice 3) Dietary diversity for children 6–23 months, with an emphasis on consumption of food sources of iron and vitamin A, and foods that enhance iron absorption**   - **Indicator 4:** Percent of children 6–23 months who ate foods from 5 or more of 8 food groups in the previous 24 hours - **Indicator 5**: Percent of children 6-23 months receiving a minimum acceptable diet - **Indicator 6:** Percent of children 6–23 months who ate iron-rich foods in the previous 24 hours - **Indicator 7:** Percent of children 6–23 months who ate vitamin A rich foods in the previous 24 hours   **(Practice 4) Optimal meal frequency for children 6–23 months of age**   - **Indicator 8:** Percent of children 6–23 months who received food the minimum acceptable number of times for their age and breastfeeding status   **(Practice 5) Early initiation of breastfeeding**   - **Indicator 9:** Percent of children 0-23 months who were put to breast within one hour of birth   **(Practice 6) Exclusive breastfeeding from birth through the first 6 months**   - **Indicator 10:** Prevalence of exclusive breastfeeding of children under six months of age   **(Practice 7) Timely introduction of appropriate complementary foods and Continued Breastfeeding**   - **Indicator 11:** Percent of children 6-8 months who received semi-solid or solid food during the previous 24 hours - **Indicator 12:** Percent of children 6-23 months who are still breastfeeding   **(Practice 8) Reduced consumption of high-calorie, low-nutrient-density (junk) food**   - **Indicator 13:** Percent of children 0-5 months and 6-23 months who consumed sugary or processed food in the previous 24 hours - **Indicator 14:** Average number of times per day children 0-5 months and 6-23 months consumed sugary or processed food - **Indicator 15:** Percent of children 0-5 months and 6-23 months who consumed tea in the previous 24 hours |
| **Module K1:** USAID Advancing Nutrition Exposure Questions |  |
| **1 - Module D:** Maternal Nutrition and Antenatal Care | **(Practice 1) Consumption of iron-folic acid (IFA) supplements by pregnant women**   - **Indicator 1:** Percent of mothers of children <2 who took iron supplements for 90 days or more during their last pregnancy - **Indicator 2:** Mean number of days on which iron tablets/syrup was taken by women (among those who took any during their most recent pregnancy) |
| **2 - Module E:** Women’s Dietary Diversity | **(Practice 2) Dietary diversity for women, with an emphasis on consumption of food sources of iron and foods that enhance iron absorption**   - **Indicator 3:** Percent of mothers of children <2 who ate foods from 5 or more of 10 food groups in the previous 24 hours |
| **3 - Module G:** Deworming | **(Practice 9) Presumptive treatment of helminth infections for pregnant women and children**   - **Indicator 16:** Percent of women who received advice to take deworming medicine during pregnancy |
| **4 - Module H:** Water, Sanitation, and Hygiene | **(Practice 10) Handwashing at five critical times: after using the latrine, after changing a baby’s diaper/cleaning a child, after handling animals, before preparing food, and before feeding a child**   - **Indicator 17:** Percent of women who practice handwashing at least three out five critical times - **Indicator 18:** Percent of households with soap and water at a handwashing station on premises |
| **5 - Module I:** Food Storage and Preservation | **(Practice 11) Adoption of methods for safe and prolonged storage of nutrient-dense produce for the winter**   - **Indicator 19:** Percent of women who stored and preserved nutrient-dense products for consumption during the last winter |
| **6 - Module J:** Television and Social Media | - **Indicator 31:** Percent of people who had seen at least one TV spot |
| **7 - Module K2: Knowledge questions** |  |
| **8 - Module L:** Gender and Decision Making | - **Indicator 20:** Percent of women reporting increased decision-making power with husband and/or family |

**Contents**

[**MODULE A: Introduction and Informed Consent**](#_heading=h.62yhrkxaoet7) **[4](#_heading=h.62yhrkxaoet7)**

[**Module C: Household Roster, Socio-economic and Demographic Data**](#_heading=h.iner5f4qmh1a) **8**

[**Module F: Children’s Nutrition**](#_heading=h.nvp779eebfd5) **18**

[**Module K1: Nutrition Exposure Questions**](#_heading=h.rpkduooa3ytf) **29**

[**Module D: Maternal Nutrition and Antenatal Care**](#_heading=h.nvp779eebfd5) **13**

[**Module E: Women’s Dietary Diversity**](#_heading=h.nvp779eebfd5) **16**

[**Module G: Deworming**](#_heading=h.46zuslqk6a2q) **23**

[**Module H: Water, Sanitation, and Hygiene**](#_heading=h.fqukh1ro1d6k) **24**

[**Module I: Food Storage and Preservation**](#_heading=h.dsc0sv5qsjlf) **26**

[**Module J: Television and Social Media**](#_heading=h.uuwbha9t5yzm) **27**

[**Module K2: Knowledge Questions**](#_heading=h.rpkduooa3ytf) **29**

[**Module L: Gender Questions**](#_heading=h.cz2sqsgh0ete) **32**

**INTRODUCTION AND INFORMED CONSENT**

# MODULE A: Introduction and Informed Consent

Greetings: **Hello, my name is______. I represent an independent research agency ____________, which conducts a survey in Batken and Jalal-Abad oblasts to learn about nutrition among women with at least one child 0-23 months old. The information we collect will help design better programs to reach women and young children.** **If you are fully interviewed, you will receive 55 KGS to your number within 48 hours. Your views are important for us. Your participation is voluntary and all of your answers will be kept strictly confidential. The survey will take about 30 minutes. Please note the interview may be recorded for quality purposes. Do you have questions?**

**A0. Operator, please indicate the gender of the respondent based on your judgments on the respondent’s voice, do not ask this question unnecessarily**

1. Man

2. Woman - GO to A3

3. Child

**A0a.** / Ask if A0 = 1,3 / **We are interviewing mothers over 18 who have at least one child between 0 and 23 months of age (up to 1 year and 11 full months old). Please hand over the phone to a woman in your household who has children from 0 to 23 months?**

OPERATOR, IF RESPONDENT ASKS WHERE WE HAVE THE RESPONDENT’S PHONE NUMBER, SAY THE FOLLOWING:

*The phone numbers were provided by the medical institutions in which you are registered at the place of residence, and where you went for medical help. To conduct this study, official permission was obtained by order of the Ministry of Health.*

DO NOT READ OUT ANSWER OPTIONS

1. Refused to hand over the phone to the woman - thank and end the interview

2. There is no woman in the house with children aged 0-23 months - thank and end the interview

3. A woman with children aged 0-23 months is now busy / not at home, ask for a convenient time for her and call the respondent

4. Asked to call back to another number

5. The phone was handed over to the woman. GO TO A0b

6. Other (specify) ________ thank and end the interview

**A0b.** Ask if A0a=5 / **Hello, my name is______. I represent an independent research agency ____________, which conducts a survey in Batken and Jalal-Abad oblasts to learn about nutrition among women with at least one child 0-23 months old. The information we collect will help design better programs to reach women and young children. If you are fully interviewed, you will receive 55 KGS to your number within 24 hours. Your views are important for us. Your participation is voluntary and all of your answers will be kept strictly confidential. The survey will take about 30 minutes. Please note the interview may be recorded for quality purposes. Do you have questions? Do you agree to take part in the survey?**

1. **Yes**
2. **No**

**A3a. /Ask if A0=2/ Do you agree to take part in the survey?**

1. **Yes** _ GO TO A1
2. **No**

**A6.** Ask if A3a=2 A0b=2 / **Could you share the numbers of mothers you know from Batken or Jalal-Abad oblasts with children under two years old? They can also receive 55 soms as a reward for completing the survey.**

- - - 1. _______________
      2. I have not such acquaintances

**A3b. /**Ask if A3a=2/ **Please indicate the reason for your refusal:**

DO NOT READ OUT THE ANSWER OPTIONS

1. Don't have time/too busy
2. No answer given/ hung up
3. Don’t want to be interviewed at all
4. I am not allowed by my relatives / I am afraid
5. Other (specify)______

**Thank the respondent and end the interview**

**A1. /***Ask if* *A3a=1,* *A0b=1, А0=2/* **What language would you prefer to use when we speak?**

1. **Kyrgyz**
2. **Russian**
3. **Other –** If the respondent does not understand both languages at all, then thank the respondent and end interview

**Name. What is your name?**

Respondent name ______________

**S2. Before beginning the main survey, I need to confirm your age. How old are you (full years)?**

Exact age in years *________________*

98. Refusal → S2b

If 18 years of age or older - Go to question A2

***S2a.*** / Ask if S2_1<18/ **Unfortunately, we survey only women above 18 years of age. Thank you. Good bye*.*** - End interview

**S2b.** / Ask if S2=98 / **Could you tell instead whether you are over or under 18?**

1. **18 or over** – Go to question A2
2. **Under 18** → S2c

**98. Refusal -** Thank the respondent and end the interview

**S2c.** / Ask if S2b=2 **/ Unfortunately, we survey only women above 18 years of age. Thank you. Good bye. -** End the interview

**A2.** / Ask If S2_1≥18, S2b=1 / **Do you have your own children 0-23 months of age (up to full 1 year and 11 months) living in your house (she herself gave birth to)?**

1. **Yes** → S3
2. **No** GO TO **А2а**

***98. Refusal*** - Thank the respondent and end the interview

**А2а.** /Ask if А2=2/ **Unfortunately, we survey only women with children 0-23 months of age. Thank you. Good bye!** - End interview

# Module C: Household Roster, Socio-economic and Demographic Data

| **QUESTIONS - MODULE C** |
| --- |
| **S3_1. In which oblast do you live / have lived for the last six months?**  *1.Batken oblast*  *2. Jalal-Abad oblast*  *3. Recently moved to another oblast, but less than 2 months ago lived in Batken oblast*  *4. Recently moved to another oblast, but less than 2 months ago lived in Jalal-Abad oblast*  *5. Another oblast - At the moment we are interviewing only those who live in Batken and Jalal-Abad oblasts, thank you, goodbye!* |
| **S3_2. In which rayon do you live / have lived for the last six months?**  Batken oblast:  1. Batken rayon  2. Leilek rayon (incl. Isfana city)  3. Kadamjay rayon (incl. Kadamjay and Aidarken cities)  4. Kyzyl-Kiya city (only Karavan, Ak-Bulak and Jin-Jigen villages)  5. Sulukta city (only pgt. Vostochnyi)  6. Batken city (only Bazar-Bashy and Bulak-Bashy villages)  Jalal-Abad oblast:  1 Aksy rayon  2. Bazar-Korgon rayon  3. Nooken rayon  4. Suzak rayon  5. Recently moved to another rayon / city, but less than 2 months ago lived in Aksy rayon  6. Recently moved to another rayon/ city, but less than 2 months ago lived in Bazar-Korgon rayon  7. Recently moved to another rayon / city, but less than 2 months ago lived in Nooken rayon  8 Moved recently to another rayon / city, but less than 2 months ago lived in Suzak rayon  9 Other rayon / city - We are not interviewing respondents from this rayon at the moment, thanks, goodbye! |
| **S3d. Indicate the village where you live / have lived for the last six months?**  OPERATOR, IF THE RESPONDENT RECENTLY LIVED IN ONE OF THE FOLLOWING VILLAGES, HOWEVER, LESS THAN 2 MONTHS AGO MOVED TO ANOTHER VILLAGE, THEN ASK WHICH VILLAGE SHE MOVED FROM AND CHOOSE THAT ONE FROM THE LIST  IF MORE THAN 2 MONTHS AGO THE RESPONDENT MOVED TO ANOTHER VILLAGE, CHOOSE FROM THE LIST OF A VILLAGE (IF THERE IS), IN WHICH SHE LIVES NOW. IF THIS VILLAGE IS NOT IN THE LIST, CHOOSE ANOTHER VILLAGE, RECORD THE VILLAGE AND END THE INTERVIEW  **2. Another village** ______________ At the moment, we are not interviewing respondents from this village, thank you, goodbye!  *(A list of settlements will be presented, distributed by each Aiyl Okmotu / Aiyl Aimak and by type of zone)* |
| S3b. *(This question is not asked by the operator, the system itself must allocate the village to a specific Ail Okmotu)*  **Aiyl Aimak / Aiyl Okmotu**  Batken oblast:  1. Kara-Bak  2. Kadamjay city  3. Uch-Korgon  4. Ak-Turpak  5. Kyzyl-Kiya city  6. Maidan  7. Halmion  8. Markaz  9. Aydarken city  10. Absamat Masaliev  11. Kara-Bulak  12. Dara  13. Jean-Ger  14. Sumbul  15. Kyshtut  16. Kotormo  17. Kulundu  18. UTS. Vostochnyi  19. Cake-Ghoul  20. Leilek  21. Ak-Sai  22. Samarkandek  23. Orozbekov  24. Ak-Tatyr  25. Beshkent  26. Isfana city  27. Ak-Suu  28. Suu-Bashy  29. Chowai  30. Council  31. Margun  32. Batken city  Jalal-Abad oblast:  1.Yrys  2. Kenesh  3. Barps  4. Arstanbap  5. Tash-Bulak  6. Bagysh  7. Kyzyl-Tuu, Suzak rayon  8. Mombekov  9. Nazaraliev  10. Mogul  11. Kyzyl-Kol  12. Maylyan  13. Shaidan  14. Akman  15. Avletim  16. Kara-Darya  17. Beshik-Jon  18. Saypidin-Atabekov  19. Kashka-Suu  20. Kurmanbek  21. Talduu-Bulak  22. Zhany-Zhol  23. Kara-Jygach  24. Aral  25. Kar-Suu  26. Massy  27.G. Kerben  28. Kyzyl-Unkur  29. Lenin  30. Kyzyl-Tuu, Aksy rayon  31. Zherge-Tal  32. Kok-art  33. Suzak  34. Kara-Alma  35. Ak-Suu  36. Ak-Zhol |
| **S3c. Intervention or comparison area**  *(This question is not asked by the operator, the system itself must allocate the type of zone to a specific Ail Okmotu)*  1. Intervention area  2. Comparison area |
| **S9. How many people live in your household, including you, all children and adults?**  1. Number of people __________ *(the value must be at least 2 people)*  98. Refusal |

| **QUESTIONS** |
| --- |
| **C0a. How many children do you personally have between the ages of 0 and 5 full months?**  (OPERATOR, CLEARLY EXPLAIN TO THE RESPONDENT THAT CHILDREN 0-5 MONTHS ARE CHILDREN, STARTING ONE DAYS OF BIRTH, ENDING TO THOSE WHO IS 5 FULL MONTHS, EVEN HE / SHE HAS ONLY ONE DAY REMAINING UNTIL 6 MONTHS)  1. 1  2. 2  3. 3  4. I have no children aged 0-5 months |
| **C0b. How many children do you personally have between the ages of 6 and 23 full months?**  (OPERATOR, CLEARLY EXPLAIN TO THE RESPONDENT THAT CHILDREN 6-23 MONTHS ARE CHILDREN, STARTING WITH THE 6TH FULL MONTHS AND ENDING TO THOSE WHO IS AT LEAST 1 DAY REMAINING UNTIL 2 YEARS OLD)  1. 1  2. 2  3. 3  4. I have no children aged 6-23 months |
| **C0c.** / Ask if C0a = 1 and C0b = 4 or C0a = 4 and C0b = 1 / **What is the name and date of birth of this child?**  OPERATOR, FOLLOWING QUESTIONS WILL BE ASKED ABOUT THIS CHILD) – GO TO C5  1. Name_______  2. Day (1 to 31) _______  3. Month (from 1 to 12) ______  4. Year (FROM 2022 TO 2022 - for children 0-5 months and FROM 2020 TO 2022 - for children 6-23 months) _______ |
| **C0d.** / Ask if С0a=1 и С0b=1,2,3 / **What is the name and date of birth of the child between 0 and 5 full months?**  OPERATOR, FOLLOWING QUESTIONS WILL BE ASKED ABOUT THIS CHILD) – GO TO C5  1. Name_______  2. Day (1 to 31) _______  3. Month (from 1 to 12) ______  4. Year (FROM 2022 TO 2022) _______ |
| **C0e.** / Ask if С0a = 2,3 and С0b = 1,2,3,4 / **What is the name and date of birth of one of your children aged 0 to 5 full months?**  OPERATOR, CHOOSE ANY ONE CHILD FROM TWINS OR TRIPLETS. ALL QUESTIONS OF THE QUESTIONNAIRE WILL BE ASKED EXACTLY FOR THIS SELECTED CHILD – GO TO С5  1. Name_______  2. Day (1 to 31) _______  3. Month (from 1 to 12) ______  4. Year (FROM 2022 TO 2022) _______ |
| **C0f**./ Ask if C0a = 4 and C0b = 2.3 / **What is the name and date of birth of one of your children aged 6 to 23 full months, whose birthday occurred most recently?**  (IF THE RESPONDENT HAS ONLY TWINS OR TRIPLETS, CHOOSE ANY ONE OF THEM FOR WHICH THE FOLLOWING QUESTIONS WILL BE ASKED IN THE QUESTIONNAIRE) - GO TO C5  OPERATOR, USE THE PAST BIRTHDAY METHOD NEAREST TO THE DATE OF THE SURVEY  1. Name_______  2. Day (1 to 31) _______  3. Month (from 1 to 12) ______  4. Year (FROM 2020 TO 2022) _______ |
| **C5**. **Is (NAME from C0c, C0d, C0e, C0f) a male or female?**   1. Male 2. Female |

| **QUESTIONS** |
| --- |
| **S5. What is your education at the moment?**  1. No education  2. Primary general education (4 classes)  3. Basic secondary (9 grades)  4. General secondary (11 grades)  5. Initial vocational education (prof. Lyceum)  6. Secondary specialized education (technical school, college)  7. Incomplete higher education (3 courses or more)  8. Higher (completed bachelor's, master's, etc.)  98. Refusal to answer [DO NOT READ] |
| **C6. What is your nationality?**   1. Kyrgyz 2. Uzbek 3. Tajik 4. Uighur 5. Dungan 6. Turkish 7. Russian 8. Tatars 9. Other (specify) _______________ |
| **S8. What is your current marital status?**   1. Married 2. Divorced 3. Not Married 4. Widower   98. Refused to answer [DO NOT READ] |

# Module F: Children’s Nutrition

| **QUESTIONS - MODULE F** |
| --- |
| **F1. Now, we would like to ask a few questions regarding your child.**  **Where did you give birth to (NAME from C0c, C0d, C0e, C0f)?**  OPERATOR, READ THE ANSWER OPTIONS IF NECESSARY   1. At home 2. Government hospital 3. Government maternity 4. Private hospital/maternity 5. Other (Specify) _____________________ |
| **F2.** **Did you ever breastfeed (NAME)?**   1. Yes 2. No SKIP → F9 |
| **F3.** /Ask if F2=1/ **How long after birth did you first put (NAME) to the breast?**  OPERATOR, IF LESS THAN 24 HOURS, RECORD HOURS. OTHERWISE, RECORD DAYS.   1. Hours ___________ 2. Days ________ 3. Within 1 hour   98. Don’t know / do not remember / difficult to answer [DO NOT READ] |
| **F4**. / Ask if F2=1/ **During the first three days after delivery, did you give (NAME) the liquid that came from your breasts, including the yellow milk?**  1. Yes  2. No  98. Don’t know / do not remember / difficult to answer [DO NOT READ] |
| **F5.** / Ask if F2=1/ **In the first three days after delivery, was (NAME) given anything to drink other than breast milk?**  1. Yes  2. No  98. Don’t know / do not remember / difficult to answer [DO NOT READ] |
| **F6.** / Ask if F2=1, F5=1/ **What liquid did you give?**  DO NOT READ THE LIST OF ANSWER OPTIONS. MARK ALL THAT APPLY.   1. Milk (other than breastmilk) 2. Plain water 3. Sugar or glucose water 4. Gripe water 5. Sugar-salt-water solution 6. Fruit juice 7. Infant formula (for instance, “malysh”, “malutka” etc. 8. Tea / infusions 9. Honey 10. Dill water 11. Other (specify)_____________________ |
| **F7.** / Ask if F2=1 / **Are you still breastfeeding (NAME)?**   1. Yes 2. No 3. Refusal [DO NOT READ] |
| **F7_1.** /Ask if F2=1, F7=2/ **What was the reason for stopping breastfeeding?**  OPERATOR, READ OUT THE ANSWER OPTIONS IF NECESSARY  1. Little or a lot of milk  2. Too liquid milk  3. Pain during feeding  4. Due to illness of the mother or her treatment with antibiotics  5. Diseases of the breast during breastfeeding (mastitis, lactostasis, etc.)  6. Milk intolerance of a child  7. The baby could not suck  8. Could not put correctly to the breast  9. Lack of support from family members  10. No time for breastfeeding, it is necessary to return to work  11. Got pregnant, therefore, I stopped the breastfeeding  12. Other (specify) _____ |
| **F8.** / Ask if F2=1, F7=2/ **For how many months did you breastfeed (NAME)?**  1. Number of months ___________  2. Less than 1 month |
| **F9. Did (NAME) drink anything from a bottle with a nipple within the last day (24 hours)?**  1. Yes  2. No  98. Don’t know / do not remember / difficult to answer [DO NOT READ] |
| F10. **Now I would like to ask you about liquids or foods (NAME) had within the last day (24 hours).**  **Did (NAME) drink the following liquids?**  READ THE LIST OF LIQUIDS (1 THROUGH 7, STARTING WITH “BREAST MILK”).  1. Breast milk  2. Animal milk  3. Plain water  4. Commercially available infant formula  5. Any fortified, commercially available infant and young child food” [e.g. Cerelac, compotes or juices from Agusha, Fruto-Nyanya, etc.]  6. Tea  7. Any sugar-sweetened beverages (tea / coffee / cocoa with sugar, jam, compotes, etc.)  8. Any other liquids (chalap, kymyz, bozo, zharma, maksym, etc.)  ANSWER ON EACH LINE:  1. Yes  2. No  98. Don’t know / do not remember / difficult to answer [DO NOT READ]  **F10_2a**. / Ask if F10_2=1/ **How many times did s/he drink animal milk within the last day (24 hours)?**  1. __________________ times  98. Don’t know / difficult to answer  **F10_4a.** / Ask if F10_4=1 / **How many times did s/he drink commercially available infant formula within the last day (24 hours)?**  1. __________________ times  98. Don’t know / difficult to answer  **F10_7a.** / Ask if F10_7=1 / **How many times did s/he drink sugar-sweetened beverages within the last day (24 hours)?**  1. __________________ times  98. Don’t know / difficult to answer |
| **F11. We would also like to know, within the last day (24 hours) if your child ate the following foods, even if it was served in combination with other foods, including in other meals. If your child ate more than one tablespoon of the following foods, indicate the answer "Yes", if less than 1 tablespoon or did not eat at all, indicate the answer "No". Do not include any food that was only eaten in very small quantities, for example mainly to add flavor.**  **Did (NAME) eat the following:**  1. Porridge (bylamyk), bread, rice, buckwheat, corn, noodles, or other foods made from grains  2. Pumpkin, carrots, squash, that are yellow or orange inside, red pepper (sweet)  3. Potatoes, turnip or any other foods made from roots  4. Any dark green leafy vegetables such as: broccoli, spinach, sorrel/dock  5. Apricot, peaches, persimmon, melon  6. Other fruits or vegetables: Apple, banana, dates, grapes, qiwi, lemon, mandarin orange, orange, pear, pineapple, plum, pomegranate (anar), cherries, raspberry, strawberry, watermelon  7. Cabbage (common and red varieties), cauliflower, celery, cucumbers, tomatoes, eggplant, green, yellow pepper, mushroom, onion, radish  8. Liver, kidney, heart, gizzard or other organ meats  9. Any meat, such as beef, goat, lamb, mutton, yak, chicken, duck, turkey, other birds  10. Chicken, quail eggs or any other bird eggs  11. Fish and seafood  12. Any foods made from beans, peas, lentils, nuts, or seeds  13. Hard and soft cheese, kefir, yoghurt/curd, suzmo  17. Ice cream, cream, sour cream (kaimak), sugar-sweetened yogurts  14. Vegetable, olive oil, fats, butter, mayonnaise or foods made with any of these  15. Any sugary foods such as chocolates, sweets, candies, pastries, cakes, or biscuits  16. Any other processed foods such as potato chips, crackers, sausages, kirieshki, etc.  ANSWER ON EACH LINE:  1. Yes  2. No  98. Don’t know / do not remember / difficult to answer [DO NOT READ]  **F11_15a.** / Ask, if F11_15=1 / **How many times within the last day (24 hours) did (NAME) eat sugary foods?**  1. __________________ times  98. Don’t know / difficult to answer [DO NOT READ]  **F11_16a.** / Ask, if F11_16=1 / **How many times within the last day (24 hours) did (NAME) eat processed foods such as potato chips, crackers, sausages, kirieshki, etc.?**  1. __________________ times  98. Don’t know / difficult to answer [DO NOT READ] |
|  |
|  |
|  |
|  |
|  |
|  |
|  |
|  |
|  |
|  |
|  |
|  |
|  |
|  |
|  |
| **F12.** / Ask if ALL “No” in F11 / **Did (NAME) eat any solid, semi-solid, or soft foods at all within the last day (24 hours)?**  FOODS MAY INCLUDE MASHED OR PUREED FOOD, ALONG WITH PORRIDGES, PAPS, THICK GRUELS, STEWS, ETC. SOLID FOODS – E. G., FAMILY FOODS – MEAT, POTATOES, AND BREAD – SHOULD ALSO BE INCLUDED ЗАЧИТАТЬ   1. Yes 2. No   98. Don’t know / difficult to answer [DO NOT READ]  **F12a.** / Ask if F12 = 1 / **What kind of foods did (NAME) eat?**  DO NOT READ OUT THE ANSWER OPTIONS, MULTIPLE ANSWER OPTIONS  1. Porridge, bread, rice, buckwheat, corn, noodles, or other foods made from grains  2. Pumpkin, carrots, squash, that are yellow or orange inside, red pepper (sweet)  3. Potatoes, turnip or any other foods made from roots  4. Any dark green leafy vegetables such as: broccoli, spinach, sorrel/dock  5. Apricot, peaches, persimmon, melon  6. Other fruits or vegetables: apple, banana, dates, grapes, qiwi, lemon, mandarin orange, orange, pear, pineapple, plum, pomegranate (anar), cherries, raspberry, strawberry, watermelon  7. Cabbage (common and red varieties), cauliflower, celery, cucumbers, tomatoes, eggplant, green, yellow pepper, mushroom, onion, radish  8. Liver, kidney, heart, gizzard or other organ meats  9. Any meat, such as beef, goat, lamb, mutton, yak, chicken, duck, turkey, other birds  10. Chicken, quail eggs or any other bird eggs  11. Fish and seafood  12. Any foods made from beans, peas, lentils, nuts, or seeds  13. Hard and soft cheese, kefir, yoghurt/curd, suzmo  18. Ice cream, cream, sour cream (kaimak), sugar-sweetened yogurts  14. Vegetable, olive oil, fats, butter, mayonnaise or foods made with any of these  15. Any sugary foods such as chocolates, sweets, candies, pastries, cakes, or biscuits  16. Any other processed foods such as potato chips, crackers, sausages, kirieshki, etc.  17. Other food (specify) _________ |
| **F13.** /Ask if at least one is Yes F11_1 - F11_17 = 1/ **How many times did (NAME) eat solid, semisolid, or soft foods other than liquids Within the last day (24 hours)? Small snacks and small feeds such as one or two bites of mother’s or sister’s food should not be counted.**  **Liquids also do not count for this question. Do not include thin soups or broth, watery gruels, or any other liquid.**  (OPERATOR, WE WANT TO FIND OUT HOW MANY TIMES THE CHILD ATE ENOUGH TO BE FULL. USE PROBING QUESTIONS TO HELP THE RESPONDENT REMEMBER ALL THE TIMES AND FOODS THE CHILD ATE WITHIN THE LAST DAY)    1. Number of times_____  98. Don’t know / do not remember [DO NOT READ] |

| **Module K1. Nutrition Exposure Questions** |
| --- |
| **K1a. Now we would like to ask a few questions about the nutritional information you have heard from various sources.**  **Over the past year, have you or your household members received any information on the following topics?**  1. Breastfeeding  2. Complementary feeding  3. Having a nutrient-rich and diverse diet  4. Ways to prevent/treat anemia  5. Hygiene, including hand washing and other health measures on sanitation  6. Nutrition for pregnant women and mothers  7. Food storage and preservation  8. Preventing intestinal worms  9. Other information about nutrition __________   - 1. Yes – IF at least one Yes - GO TO K1b   **K1a_9a. What other nutritional information have you or your household members received over the past year?**  **1.____________________________** |
| **K1b. /** IF at least one Yes on K1a/ **What is the main source of information on the topic ...?**  (ASK FOR EACH TOPIC SELECTED IN K1a)  1. Breastfeeding  2. Complementary feeding  3. Having a nutrient-rich and diverse diet  4. Ways to prevent/treat anemia  5. Hygiene, including hand washing and other health measures on sanitation  6. Nutrition for pregnant women and mothers  7. Food storage and preservation  8. Preventing intestinal worms  9. Other information about nutrition _____________  CHOOSE ONE ANSWER OPTION, READ OUT THE OPTIONS ONLY IF NECESSARY   1. Household visit from community worker/ health worker 2. From social workers / activists via WhatsApp or ZOOM 3. Community meeting/community or city event 4. Visit to health facility 5. Friend or neighbor 6. Relatives 7. Social networks (e.g. Facebook, Youtube, Insagram, VKontakte, etc.) and other internet sources/websites 8. Messengers (e.g. WhatsApp, Telegram, etc.) 9. Radio 10. Television 11. Other |
| **K1c. /** IF at least one Yes on K1a/ **In what format did you receive information on the topics you mentioned?**  READ OUT THE ANSWER OPTIONS. MULTIPLE ANSWER  1. Picture  2. Video  3. Handouts / Booklets / Flyers  4. Posts or articles on social networks  5. Orally  6. Other (specify) ______ |
| **K1d. /** IF at least one Yes on K1a/ **Have you shared information with anyone on these topics?**  1. Yes (what topics?) ___________________  2. No SKIP → K2_1  98. Don't know SKIP → K2_1 |
| **K1e.** / Ask if K1d=1/ **With whom did you share your knowledge?**  DO NOT READ THE LIST, BUT MARK ALL THAT APPLIES   - 1. With her husband   2. With children   3. With the parents / parents of the husband   4. With brothers / sisters   5. With other relatives living in your home   6. With neighbors   7. With friends / girlfriends   8. With colleagues   9. Other (specify) __________ |

| **K3.** **Have you ever heard of the USAID Advancing Nutrition project?**  1. Yes  2. No  98. Do not know |
| --- |

# Q1. DO NOT READ OUT. OPERATOR, CHECK THE FIRST OPTION (IN QUEUE, NOT BY NUMBERING), WHICH IS IN FIRST PLACE: */ Randomization of options /*

# 1. 1,2,3,4,5,6

# 2. 3,4,5,6,7,8

# 3. 1,2,3,4,7,8

# 4. 1,2,5,6,7,8

# */Ask if Q1=1, 3, 4/* Module D: Maternal Nutrition and Antenatal Care

| **QUESTIONS - MODULE D** |
| --- |
| **D1. During your pregnancy with (NAME OF CHILD) did you ever visit a health facility (polyclinic, private or public clinics) for information or services related to your pregnancy?**   1. Yes 2. No   98. Don’t know/ don’t remember [DO NOT READ] |
| **D2.** / Ask if D1=1/ **How many times did you visit the health facility?**  OPERATOR, IF THE RESPONDENT DOES NOT REMEMBER THE EXACT NUMBER OF TIMES USE PROBING QUESTIONS TO HELP HER REMEMBER THE APPROXIMATE NUMBER OF TIMES  1. Number of times ___________  98. Don’t know/ don’t remember [DO NOT READ] |
| **D3.** / Ask if D1=1/ **On what week of your (NAME OF CHILD) pregnancy (number of the week) did you have your first visit at a health facility about your pregnancy?**  OPERATOR, IF ANSWER IS GIVEN IN MONTHS, CONVERT TO WEEKS FROM CALCULATION 1 MONTH = 4 WEEKS  1. Number of weeks _______________  98. Don’t know/don’t remember [DO NOT READ] |
| **D4. During your pregnancy with (NAME OF CHILD) were you prescribed or bought drugs containing iron, folic acid or other multiple micronutrients?**  OPERATOR, IF YOU ARE ASKED TO GIVE AN EXAMPLE, LIST THE FOLLOWING NAMES OF TABLETS: FERNIXIL, FERSINOL, GINO-TARDIFERON, TARDIFERON, MALTOFER FOL, FERRUM LEK, FERRO-GRADIMET, ELEVIT, ETC. USE THE GIVEN CARD WITH THE IMAGE OF THESE PREPARATIONS TO DESCRIBE THE PACKAGING TO THE RESPONDENT IF NECESSARY.   1. Yes 2. No SKIP → D8   98. Don’t know/ don’t remember SKIP → D8 |
| **D5**. / Ask if D4=1/ **Which form of iron supplement did you receive or purchase the most of?**  CHOOSE ONE ANSWER   1. Iron tablets (Fersinol, Ferrum-Lek, Tardiferon, Ferifol, Maltofer, Ferrus, Orofer, etc.) 2. IFA tablets (Gino-Tardiferon, Yodofol, Maltofer-FOL, Folievaya Kislota, Ferrus-S, Airokeir-M, Emfetal etc.) 3. Iron syrup-like preparation (Ferrum-Lek, Fersinol, Ferrotsit, Ferrus, Orofer, Gematonic, Maltofer, Totema etc.) 4. Complex vitamins (multivitamins) (Elevit, Alfavit, Duovit, Pikovit, Rotovit, Vitrum, Emfetal, etc.) 5. Other (capsules, drops, etc.) specify _________________________   98. Don't know / Don't remember [DO NOT READ OUT] |
| **D6**. /Ask if D4=1/ **How many days in total did you take them during your pregnancy?**  IF ANSWER IS NOT NUMERIC (LONG, SEVERAL TIMES, ETC.), PROBE FOR APPROXIMATE NUMBER OF DAYS.  1. Number of days _______  98. Don’t know/ don’t remember [DO NOT READ OUT] |
| **D7.** / Ask if D4=1/ **Where did you buy or receive them?**  CHECK ALL THAT APPLY   1. Antenatal visit to health facility 2. Pharmacy 3. Other (Specify)_______________________   99. Don’t know/ don’t remember [DO NOT READ OUT] |
| **D8. Were you counseled from medical specialists on any of the following issues?**  READ LIST AND MARK ALL THAT APPLY  (OPERATOR, USE THE GIVEN CARDS IN THE CASE OF THE RESPONDENT CLARIFYING QUESTIONS WITH SYMPTOMS AND EXAMPLES OF DANGERS)  1. Danger signs during pregnancy  *[Examples for interviewer:*  *- Severe headache, dizziness, visual impairment (blurring, flickering "flies" in front of the eyes), nausea, vomiting*  *- Sudden or rapidly increasing swelling of the face and body, reducing the amount of urine*  *Seizures, loss of consciousness*  *- Bright red blood-stained vaginal discharge*  *- Sharp, strong, constant stomach pain; weakness and dizziness*  *Rapid infertility water outflow*  *- High body temperature, heartbeat, shortness of breath]*  2. Women’s diet during pregnancy  3. Rest during pregnancy  4. Self-care during pregnancy (wash, swim, use hygiene products, etc.)  5. Use of iron-containing preparations  6. Taking drugs to prevent intestinal worms  7. Birth preparedness  8. Postpartum family planning  9. Postpartum danger signs for the mother  10. Dangerous signs for a newborn  11. Breastfeeding  12. Introduction of solid and semi-solid foods for your baby |

# /*Ask if* *Q1=1,3,4/* Module E: Women’s Dietary Diversity

| **QUESTIONS - MODULE E** |
| --- |
| **E1. Now we would like to know about all the foods that you ate during the last day (within the last 24 hours) including in combination with other foods. Now I will list food items, if you ate this or that product more than one tablespoon, indicate the answer "Yes", if less than one tablespoon or if you did not eat the named product at all, indicate the answer "No".**  OPERATOR, CHECK ALL FOOD ITEMS EVEN IF THEY WERE COMBINED WITH OTHER FOODS. FOR EXAMPLE, IF THE RESPONDENT HAD A SOUP MADE WITH CARROTS, POTATOES AND MEAT, YOU SHOULD MARK “YES” FOR EACH OF THESE INGREDIENTS WHEN YOU READ THE LIST. HOWEVER, IF THE RESPONDENT CONSUMED ONLY THE BROTH OF A SOUP, BUT NOT THE MEAT OR VEGETABLE, DO NOT MARK “YES” FOR THE MEAT OR VEGETABLE.  1. Any food made from grains, like: porridge, bread, rice, buckwheat, corn, pasta/noodles or other foods made from grains |
| 2. Pumpkin  3. Carrots  4. Squash  5. Red pepper (sweet) |
| 6. Potatoes  7. Turnip |
| 8. Any dark green leafy vegetables, such as: Broccoli, spinach, sorrel/dock |
| 9. Apricot  10. Peaches  11. Persimmon  12. Melon |
| 13. Any other fruits such as: Apple, banana, dates, grapes, qiwi, lemon, mandarin orange, orange, pear, pineapple, plum, pomegranate (anar), cherries, raspberry, strawberry, watermelon |
| 14. Any other vegetables such as: Cabbage (common and red varieties), cauliflower, celery, cucumbers, tomatoes, eggplant, green, yellow pepper, mushroom, onion, radish |
| 15. Liver, kidney, heart, gizzard or other organ meats |
| 16. Beef  17. Horse  18. Lamb/Mutton  19. Chicken  20. Duck, turkey, goat, yak and other meat products |
| 22. Chicken, quail eggs or other bird eggs |
| 23. Fish and seafood |
| 24. Beans  25. Peas  E1_26. Lentils |
| 27. Any nuts such as: almond, hazelnut, pistachio, cashew, groundnut/peanut  28. Any seeds: melon, pumpkin, sesame, sunflower seeds |
| 29. Milk (including milk with water or in porridge)  30. Hard and soft cheeses  31. Kefir, yoghurt/curd, airan  32. Butter, ice cream, cream, sour cream (kaimak), tea, coffee or cocoa with milk, sugar-sweetened beverages and yogurts  33. Kymyz, chalap or suzmo  ANSWER FOR EACH LINE:  1. Yes  2. No  98. Don’t know / do not remember / difficult to answer [DO NOT READ] |

# /Ask if *Q1=1,2,3/* Module G: Deworming

| **QUESTIONS - MODULE G** |
| --- |
| **G1. Have you ever received advice or recommendations from anyone about taking deworming medicine during pregnancy?**  1. Yes  2. No  98. Don’t know / do not remember [DO NOT READ] |
| **G2.** / Ask if G1=1 / **Where did you get the information on taking deworming medicine?**  MULTIPLE ANSWER OPTIONS   1. Health provider 2. Friends or neighbors 3. Relatives 4. Internet 5. Radio 6. TV 7. Other (specify)_______ |

# /Ask if *Q1=1,2,3/* Module H: Water, Sanitation, and Hygiene

| **QUESTIONS - MODULE H** |
| --- |
| **H1_1.** **Now we’d like to ask you about water, sanitation and hygiene in your household.**  **What kind of handwashing stations does your house have?**  READ OUT ALL THE ANSWERS. MARK ALL THAT APPLY  1. Washbasins, hanging bucket or other portable equipment  2. Jug with a basin  3. Crane sink, column or other fixed installation  4. Other (specify)____________________  98. Don't know / Difficult to answer [DO NOT READ] |
| **H1_2.** **Does at least one of the household handwashing stations have both soap and water available?**  1. Yes  2. No  98. Don’t know / difficult to answer [DO NOT READ] |
| **H2a**. **When do you usually wash your hands?**  OPERATOR, DO NOT READ OUT THE ANSWER OPTIONS, MARK ALL THAT APPLY. IF ANSWERED ALWAYS, ASK IN WHAT EXACTLY CASES SHE WASHES HANDS ALWAYS   1. Never SKIP → I1 2. Before food preparation 3. Before feeding children 4. Before eating 5. After using the toilet 6. After cleaning defecation of a child 7. After handling livestock 8. Other (specify)_____________ 9. Other (specify)_____________ 10. Other (specify)_____________ |
| **H3.** ONLY FOR CASES MARKED IN H2a  **How often do you wash your hands…? Rarely, often or always?**  1)…before food preparation?  2) …before feeding children?  3)... before eating?  4) …after using the toilet?  5) …after cleaning the defecation of a child?  6) … after handling livestock?  7) …other   1. Rarely 2. Often 3. Always |
| **H4. When you wash your hands, how often do you use soap?**  READ ALL ANSWER OPTIONS   1. Never 2. Rarely 3. Often / Most of the time 4. Always |

**Ask if *Q1=1,2,4/* Module I: Food Storage and Preservation**

| **QUESTIONS - MODULE I** |
| --- |
| **I1.** **Did you PRESERVE (for example, by conserving, salting, making jams, salads, compotes, etc.) or STORE (by keeping products fresh) any food for consumption during the last winter?**   1. Yes 2. No   98. Don’t know |
| **I2.** Ask if I1=1/ **What methods of preserving and storing products did you use last winter?**  READ ALL ANSWERS, MARK ALL THAT APPLY   1. Sun drying or other drying 2. Boiling 3. Canning 4. Salting 5. Sugaring (jam making) 6. Pickling 7. Freezing 8. Buried under dirt or straw 9. Buried in deep trench with dirt and straw covering 10. In a cellar under the house 11. In a cold room or building 12. Other (specify) ___________ |
| **I3.** / Ask if I1=1/ **Which of the following products did you store or preserve for consumption during the last winter?**  READ ALL ANSWERS, MARK ALL THAT APPLY   1. Persimmons or Apricots 2. Carrots or Pumpkin 3. Apples, peaches, pears, quinces, pomegranate, plum or cherry / sweet cherry 4. Bell peppers 5. Cabbages 6. Onion 7. Potatoes 8. Beetroot 9. Broccoli, corn, peas and other vegetables 10. Strawberry and/or raspberry 11. Tomatoes, cucumbers 12. Other (specify)_____ |

# /Ask if *Q1=1,2,4/* Module J: Television and Social Media

| **QUESTIONS - MODULE J** |
| --- |
| **J1. Do you watch television?**  1. Да  2. Нет |
| **J2.** / Ask ifJ1=1/ **What times of day do you watch television most often?**  CHECK ONE   - 1. Morning   2. Mid-day   3. Afternoon   4. Evening   5. Late night |
| **J3.** / Ask ifJ1=1/ **What 3 channels do you watch most often?**  MARK UP TO THREE OPTIONS   1. KTRK 2. KTRK-Balastan 3. KTRK-Sport 4. KTRK-Ala-Too 24 5. KTRK-Muzyka 6. KTRK-Madaniyat 7. Ilim-bilim 8. Batken-TV 9. 5 kanal 10. 7 kanal 11. Asia TV 12. Ayan TV 13. ELTR 14. Exo Manasa 15. NTS+NTV 16. Keremet 17. Yntymak 18. Kyrgyzstan 19. MIR/MIR-24 20. NUR 21. ORT 22. RTR 23. Osh Pirim 24. Osh TV 25. Piramida 26. Region 27. TNT 28. JTR (Jalal-Abad TV) 29. Other (Specify) |
| **J4.** / Ask if J1=1/ **Have you seen at least one television spot focused on Nutrition and Hygiene messages on Batken TV, Yntymak or JTR (Jalal-Abad TV) within the previous 3 months?**   1. Yes 2. No   98. Don’t know / difficult to answer [DO NOT READ] |
| **J5.** **Do you use a Smartphone (with access to the Internet)?**   1. Yes 2. No SKIP → K1a |
| **J6.** / Ask if J5=1/ **What social media and messengers do you use?**  MARK ALL THAT APPLY   1. WhatsApp 2. Instagram 3. You Tube 4. Facebook 5. Odnoklassniki 6. Vkontakte 7. Other (specify)_______________________ |

# /Ask if *Q1=2,3,4*/ Module K2: Knowledge questions

| **QUESTIONS - MODULE K** |
| --- |
| **K2_1. Now we’d like to ask you some questions about different aspects about nutrition, please give the best answer you can.**  **Until what age should a baby receive only breast milk (nothing other than breast milk / formula)?**  OPERATOR, DO NOT READ THE ANSWER OPTIONS   - 1. Less than 5 months (1,2,3 or 4 full months, or 5 not full months)   2. Until 6th months (5 full months)   3. More than 6 months (≥6 months)   4. Other (specify) _________________   98. Do not know  **K2_2.** **At what age should soft, semi-solid foods be introduced to complement breast milk?**  OPERATOR, DO NOT READ THE ANSWER OPTIONS  1. Earlier than at 6 months  2. At 6 months  3. Starting from 7 months  4. Other (Specify) _______________  98. Do not know  **K2c. Yellow/orange/red fruits and vegetables are important sources of what vitamin?**  OPERATOR, DO NOT READ THE ANSWER OPTIONS, MULTIPLE ANSWER OPTION   1. Vitamin A 2. Other (specify) _________________   98. Do not know  **K2d. Have you ever heard of anemia?**   1. Yes 2. No   **K2e.** */Ask if K2d=1/* **What should be done to prevent anemia in women?**  OPERATOR, DO NOT READ THE ANSWER OPTIONS. AFTER GETTING A RESPONDENT'S ANSWER, REPEAT THE QUESTION TO CHECK OUT WHAT ELSE IS KNOWN BY THE RESPONDENT  MARK ALL THAT APPLIES  1. To eat iron-rich foods (liver, kidneys, heart, stomach and other internal organs: beef, horse meat, lamb, chicken, duck, turkey, goat, yak, fish and other meat products)  2. To take other fortified foods that increase hemoglobin  3. To take iron/multi-supplements or other meditation that increase the hemoglobin  4. To take medications for the treatment and prevention of worms  5. To practice proper hygiene  6. Do not drink tea (black / green)  7. Other (specify) __________  99. Do not know  **K2f. When is it necessary to wash hands?**  OPERATOR, DO NOT READ THE ANSWER OPTIONS. AFTER GETTING A RESPONDENT'S ANSWER, REPEAT THE QUESTION TO CHECK OUT WHAT ELSE IS KNOWN BY THE RESPONDENT  MARK ALL THAT APPLIES   1. Before food preparation 2. Before feeding children 3. Before eating 4. After using the toilet 5. After cleaning defecation of a child 6. Other (READ) ______   98. Do not know  **K2g. When pregnant, should women eat more, less or the same amount of food as her usual amount?**  OPERATOR, DO NOT READ THE ANSWER OPTIONS  1. Less  2. The same  3. More  98. Do not know  **K2h. Have you heard about intestinal worms?**   1. Yes 2. No     **K2j. /Ask if K2h=1/ What can you do to prevent intestinal worms?**  OPERATOR, DO NOT READ THE ANSWER OPTIONS. AFTER GETTING A RESPONDENT'S ANSWER, REPEAT THE QUESTION TO CHECK OUT WHAT ELSE IS KNOWN BY THE RESPONDENT  MARK ALL THAT APPLIES   1. Wash hands with soup 2. Wash vegetables and fruits well with boiled water before consuming/cooking 3. Cook meat and fish thoroughly 4. Use proper toilet 5. Take medications to prevent intestinal worms 6. Boil water for drinking 7. Give a deworming medicine to pet and livestock annually 8. Other (specify) ____________   98. Do not know |

# /Ask if *Q1=2,3,4*/Module L: Gender Questions

| **QUESTIONS - MODULE L** |
| --- |
| **L1.** */Ask if K2_2=1 and F2=1/* **Now we would like to ask a few questions about decision-making in your household.**  **In this (2022) or last (2021) year have you or other family members made decisions about whether to exclusively breastfeed your child (NAME)?**  READ OUT ANSWER OPTIONS  1. Yes, decisions on this issue were made this year  2. Yes, decisions on this issue were made both this and last year  2. No, decisions on this issue were made last year  3. No, decisions on this issue were not made either this year or last year  99. Difficult to answer [DO NOT READ OUT]  **L1b.** */Ask if L1=1,2/* **And did you express your personal opinion in your family when you made decisions on this issue this year (2022)?**   1. Yes 2. No 3. Not applicable, there was no need for this, since I alone make decisions on this issue [DO NOT READ OUT]   **L1a.** */Ask if L1b=1/* **To what extent do you think your opinion and words were taken into consideration this year (2022)?** READ OUT THE ANSWER OPTIONS  1. None of my opinions was taken into consideration  2. Only a few of my opinions were taken into consideration  3. Most of my opinions were taken into consideration  4. My opinions have been fully taken into consideration  99. Difficult to answer/not sure (DO NOT READ OUT)  **L2.** */Ask if L1b=1 and L1=2/* **And compared to the previous year, this year has your opinion been taken into consideration more when making decisions on this issue (*exclusively breastfeeding your child)*?**   1. Yes 2. No   **L2a.** */ Ask if L2 = 1 /* **A little more or much more have been taken into account?** READ OUT THE ANSWER OPTIONS  1. A little more  2. Much more  99. Difficult to answer (DO NOT READ OUT)  **L2b.** */ Ask if L2 = 2 /* **Less has been taken into account or remained the same?** READ OUT THE ANSWER OPTIONS  1. Less taken into account  2. Remained the same  99. Difficult to answer (DO NOT READ OUT)  **L3. In this (2022) or last (2021) year have you or other family members made decisions about whether to introduce solid and semi-solid complementary foods to your child (NAME)?**  READ OUT ANSWER OPTIONS  1. Yes, decisions on this issue were made this year  2. Yes, decisions on this issue were made both this and last year  2. No, decisions on this issue were made last year  3. No, decisions on this issue were not made either this year or last year  99. Difficult to answer [DO NOT READ OUT]  **L3b***./Ask if L3=1,2/* **And did you express your personal opinion in your family when you made decisions on this issue this year (2022)?**   1. Yes 2. No 3. Not applicable, there was no need for this, since I alone make decisions on this issue   **L3a.** */Ask if L3b=1/* **To what extent do you think your opinion and words were taken into consideration this year (2022)?** READ OUT THE ANSWER OPTIONS  1. Not taken into consideration at all  2. Only a few of my opinions were taken into consideration  3. Most of my opinions were taken into consideration  4. My opinions have been fully taken into consideration  99. Difficult to answer/not sure (DO NOT READ OUT)  **L4.** */Ask if L3b=1 and L3=2/* **And compared to the previous year, this year has your opinion been taken into consideration more when making decisions on this issue (*introducing solid and semi-solid complementary foods to your baby)?***   1. Yes 2. No   **L4a.** */ Ask if L4 = 1 /* **A little more or much more have been taken into account?** READ OUT THE ANSWER OPTIONS  1. A little more  2. Much more  99. Difficult to answer (DO NOT READ OUT)  **L4b.** */ Ask if L4 = 2 /* **Less has been taken into account or remained the same?** READ OUT THE ANSWER OPTIONS  1. Less taken into account  2. Remained the same  99. Difficult to answer (DO NOT READ OUT)  **L5. In this (2022) or last (2021) year have you or other family members made decisions about the need for both soap and water in handwashing facilities in your household?** READ OUT THE ANSWER OPTIONS  READ OUT ANSWER OPTIONS  1. Yes, decisions on this issue were made this year  2. Yes, decisions on this issue were made both this and last year  2. No, decisions on this issue were made last year  3. No, decisions on this issue were not made either this year or last year  99. Difficult to answer [DO NOT READ OUT]  **L5b. /***Ask if L5=1,2/* **And did you express your personal opinion in your family when you made decisions on this issue this year (2022)?**   1. Yes 2. No 3. Not applicable, there was no need for this, since I alone make decisions on this issue (DO NOT READ OUT)   **L5a. /***Ask if L5b=1/* **To what extent do you think your opinion and words were taken into consideration this year (2022)?** READ OUT THE ANSWER OPTIONS  1. Not taken into consideration at all  2. Only a few of my opinions were taken into consideration  3. Most of my opinions were taken into consideration  4. My opinions have been fully taken into consideration  99. Difficult to answer/not sure (DO NOT READ OUT)  **L6. /***Ask if L5b=1 and L5=2/* **And compared to the previous year, this year has your opinion been taken into consideration more when making decisions on this issue *(having soap and water available at handwashing stations within the household)*?**   1. Yes 2. No   **L6a.** */ Ask if L6 = 1 /* **A little more or much more have been taken into account?** READ OUT THE ANSWER OPTIONS  1. A little more  2. Much more  99. Difficult to answer (DO NOT READ OUT)  **L6b.** */ Ask if L6 = 2 /* **Less has been taken into account or remained the same?** READ OUT THE ANSWER OPTIONS  1. Less taken into account  2. Remained the same  99. Difficult to answer (DO NOT READ OUT)  **L7. In this (2022) or last (2021) year have you or your family members made decisions about the need to purchase nutritious foods (containing various vitamins, except sugar-sweetened and processed foods) for consumption within your household?**  READ OUT ANSWER OPTIONS  1. Yes, decisions on this issue were made this year  2. Yes, decisions on this issue were made both this and last year  2. No, decisions on this issue were made last year  3. No, decisions on this issue were not made either this year or last year  99. Difficult to answer [DO NOT READ OUT]  **L7b.** */Ask if L7=1,2/* **And did you express your personal opinion in your family when you made decisions on this issue this year (2022)?**   1. Yes 2. No 3. Not applicable, there was no need for this, since I alone make decisions on this issue [DO NOT READ OUT]   **L7a.** */Ask if L7b=1/* **To what extent do you think your opinion and words were taken into consideration this year (2022)?** READ OUT THE ANSWER OPTIONS  1. Not taken into consideration at all  2. Only a few of my opinions were taken into consideration  3. Most of my opinions were taken into consideration  4. My opinions have been fully taken into consideration  99. Difficult to answer/not sure (DO NOT READ OUT)  **L8b.** */Ask if L7b=1 and L7=2/* **And compared to the previous year, this year has your opinion been taken into consideration more when making decisions on this issue *(need of procuring nutritious foods for consumption within the household)*?**   1. Yes 2. No   **L8a.** */ Ask if L8 = 1 /* **A little more or much more have been taken into account?** READ OUT THE ANSWER OPTIONS  1. A little more  2. Much more  99. Difficult to answer (DO NOT READ OUT)  **L8b.** */ Ask if L8 = 2 /* **Less has been taken into account or remained the same?** READ OUT THE ANSWER OPTIONS  1. Less taken into account  2. Remained the same  99. Difficult to answer (DO NOT READ OUT) |

T1. /*Ask if S3_1=1, 3/* **Did the recent events on Kyrgyz-Tajik border affect your permanent residence status?**

1. Yes
2. No

T2. */Ask if S3_1=1, 3*/ **Has the food consumption of your household changed due to the recent events on the border?**

1. Yes, it has changed for the better

2. Yes, it has changed for the worse

3. No, nothing has changed

A5. **Tell me, to which number would it be more convenient for you to load units for reward for successfully completing the survey?**

**1._____________**

2. This number

**THIS CONCLUDES OUR INTERVIEW, THANK YOU FOR YOUR TIME!**

**Supplementary Material 4: Charts showing Difference in Differences Results**

**
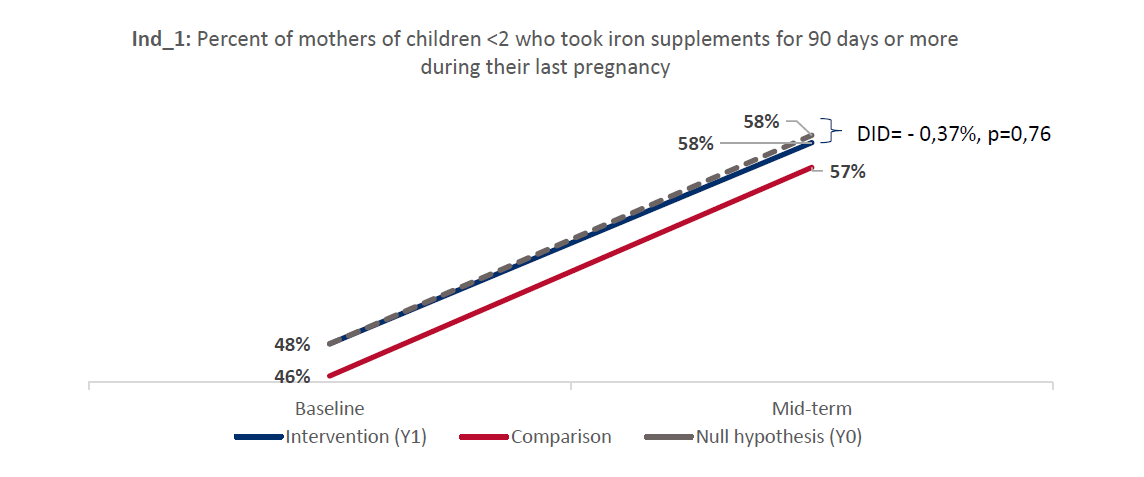
**

**
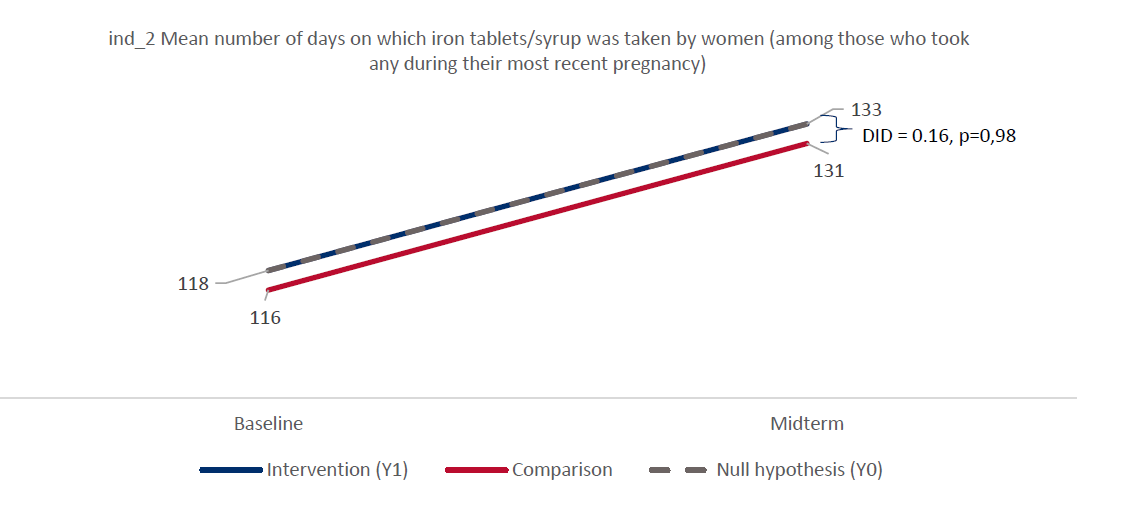
**


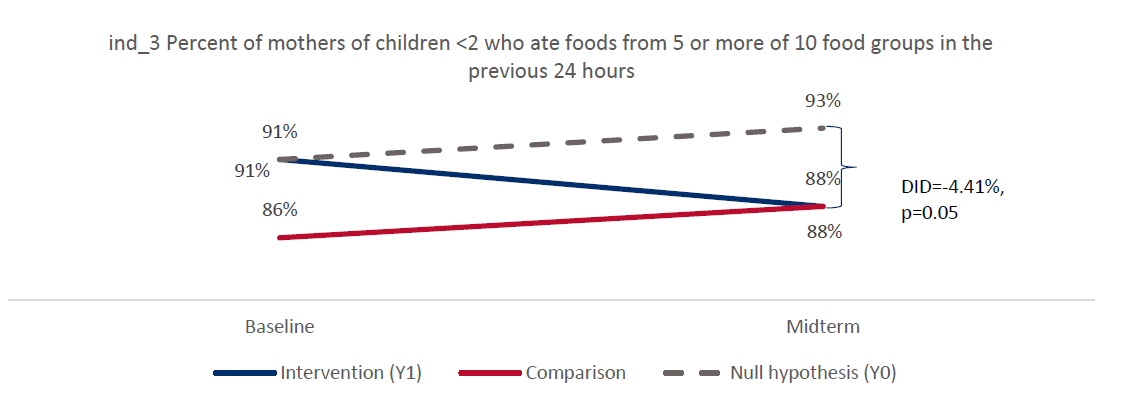


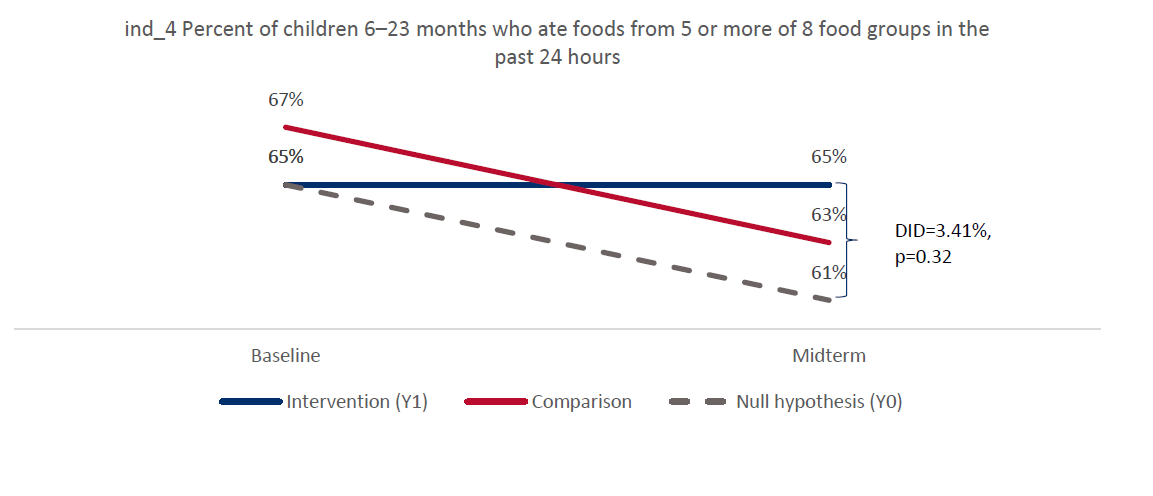


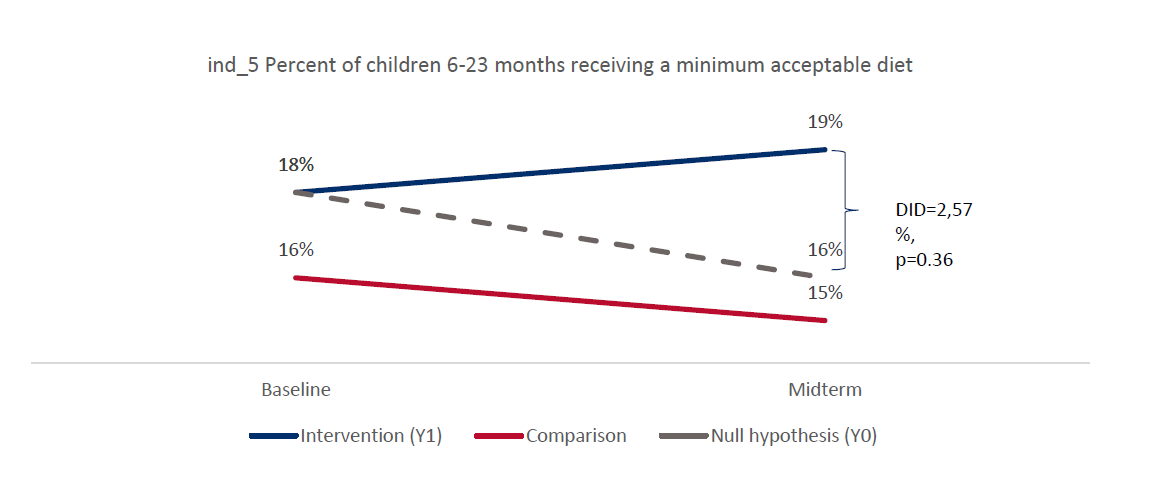


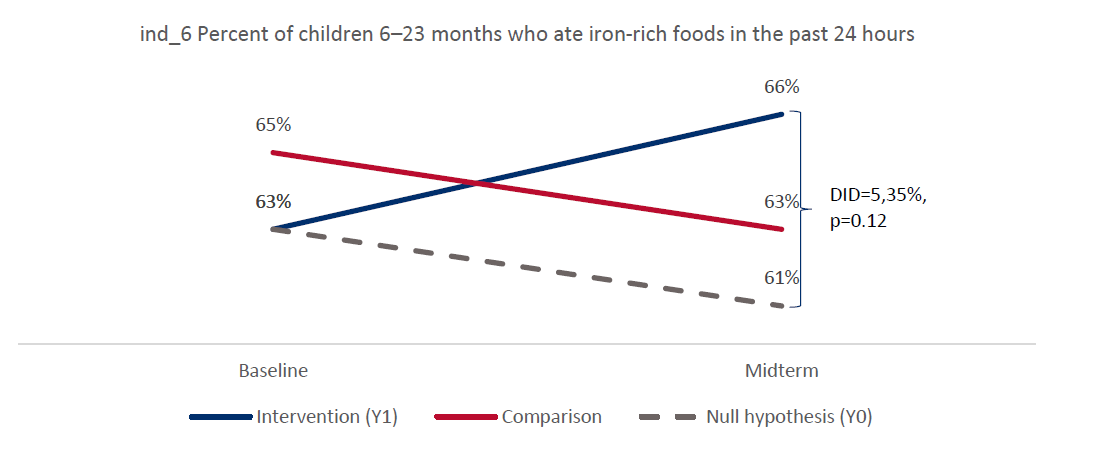


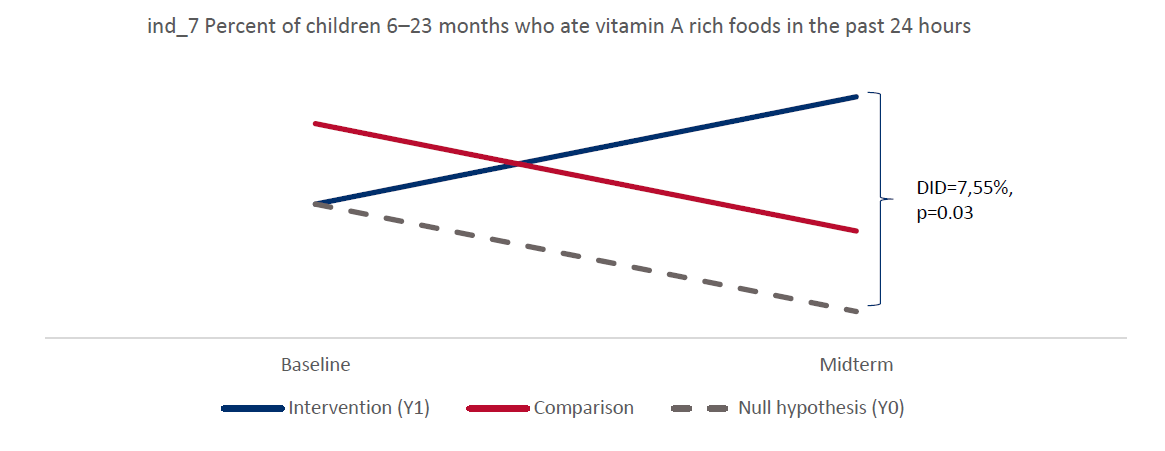


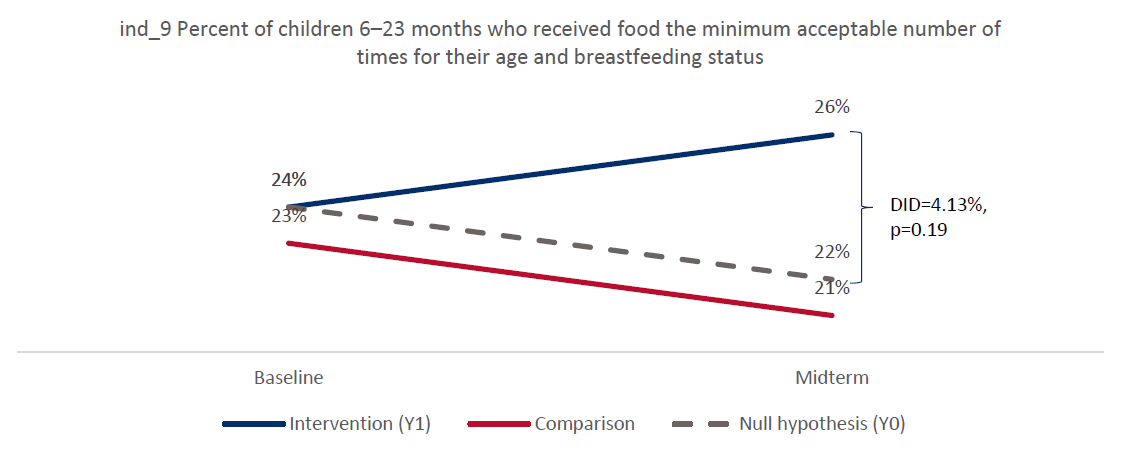


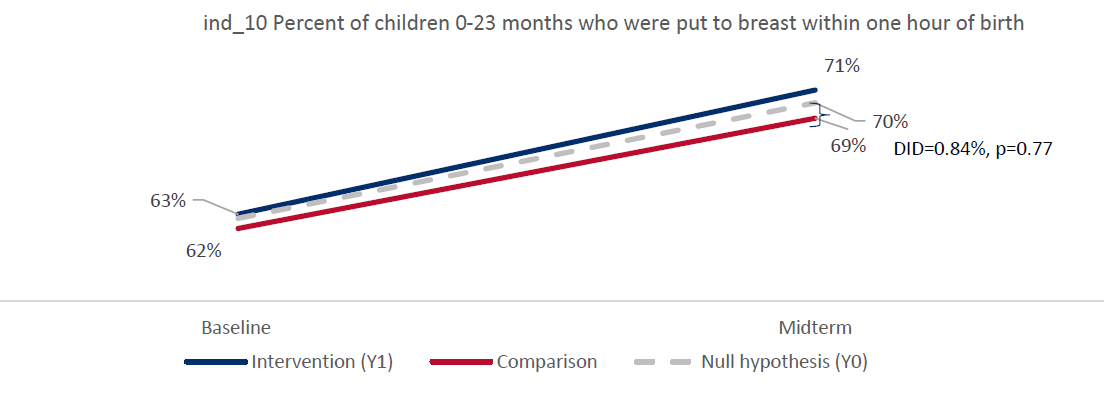


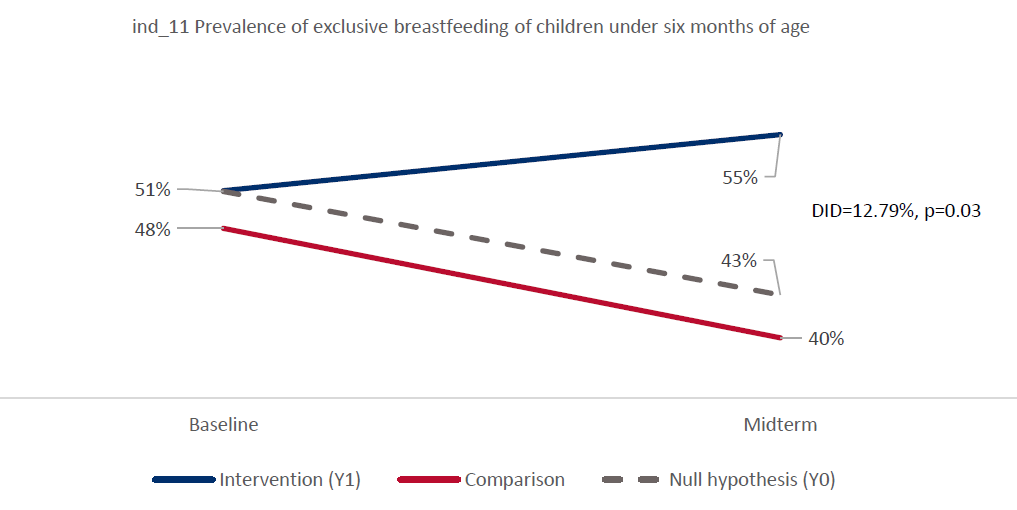


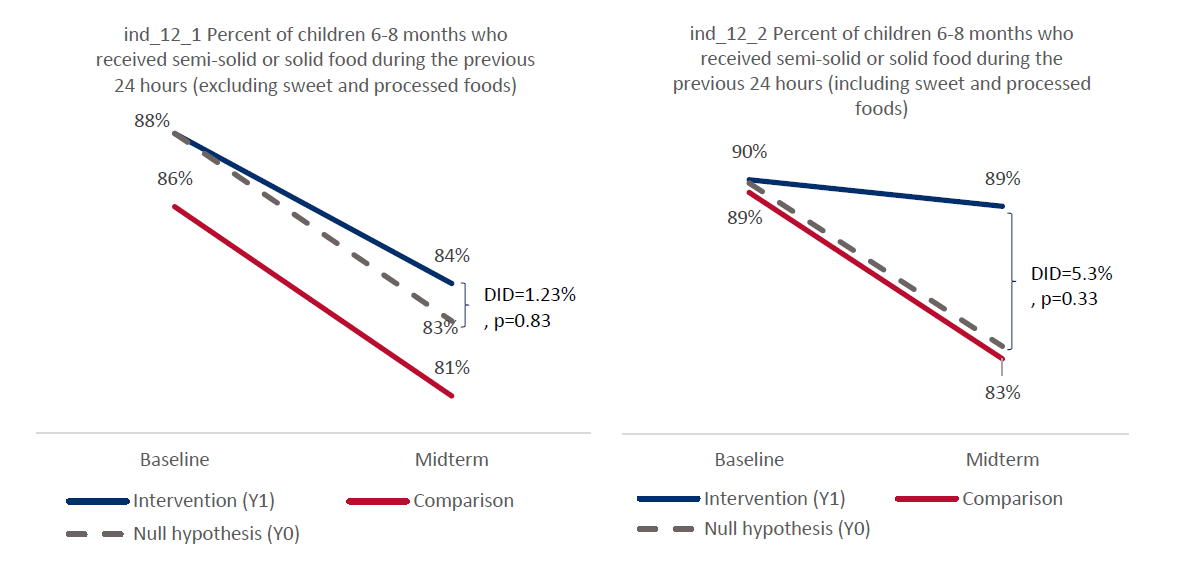


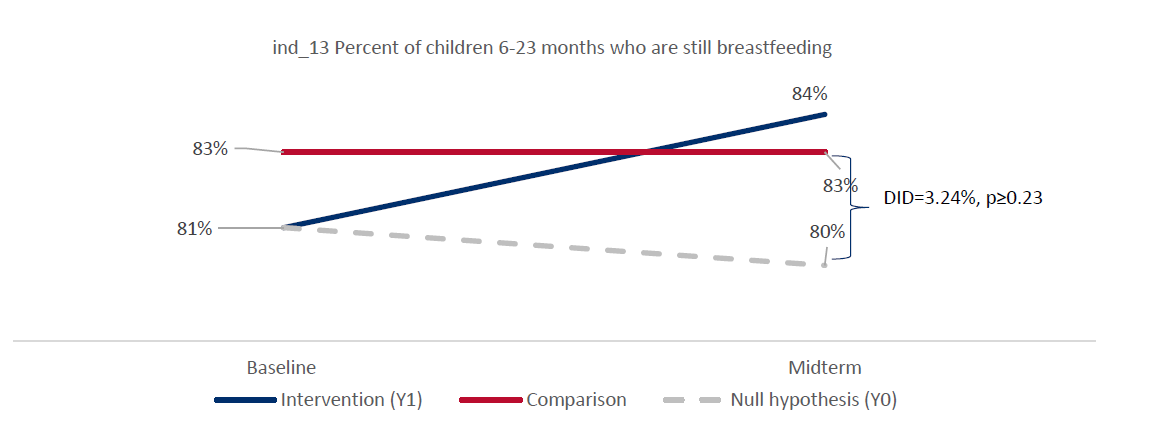


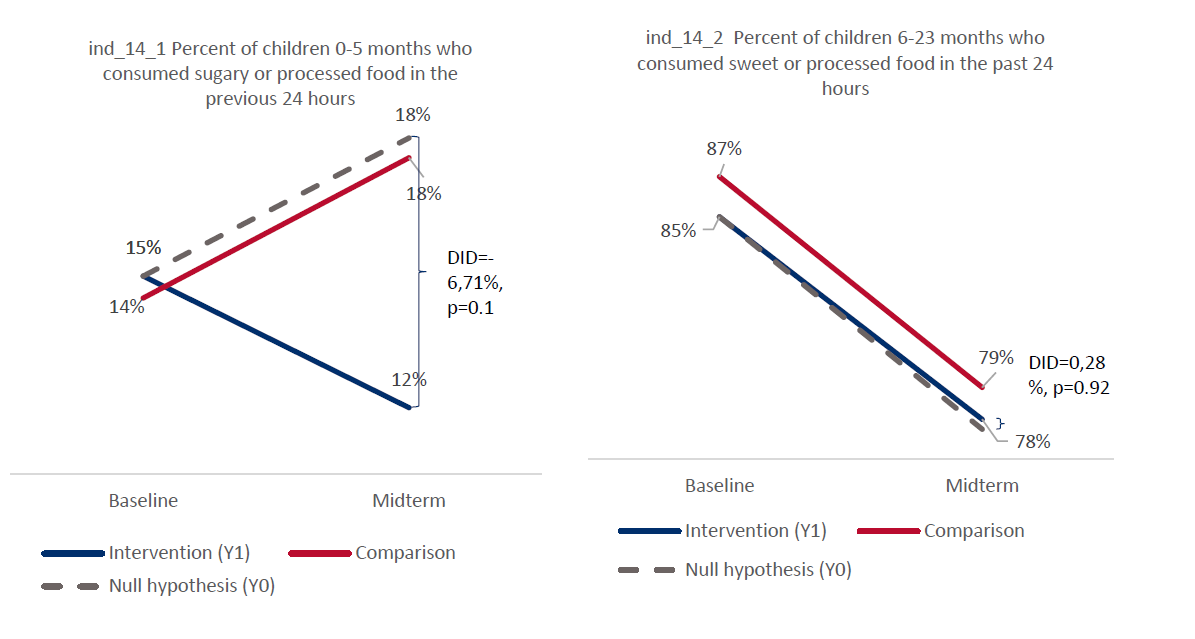


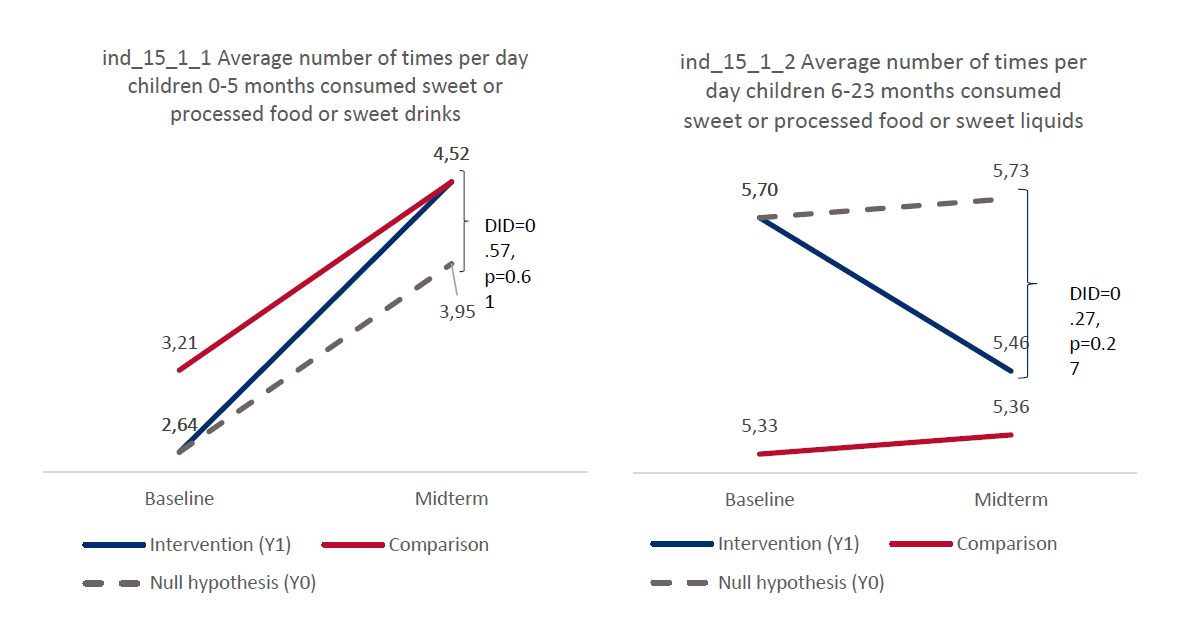


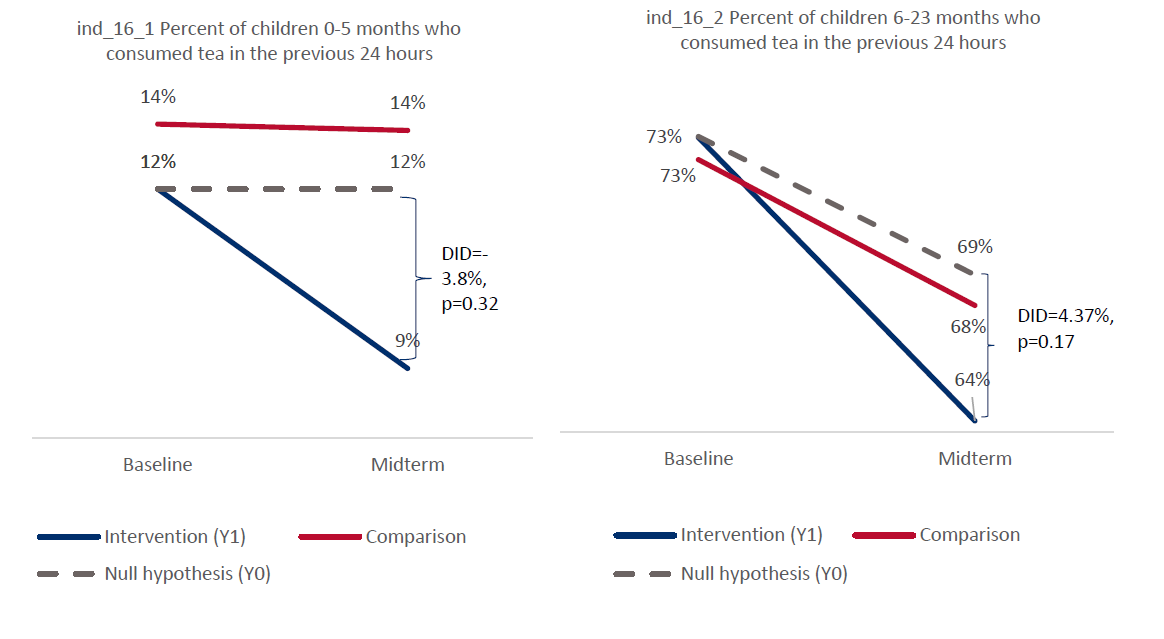


**
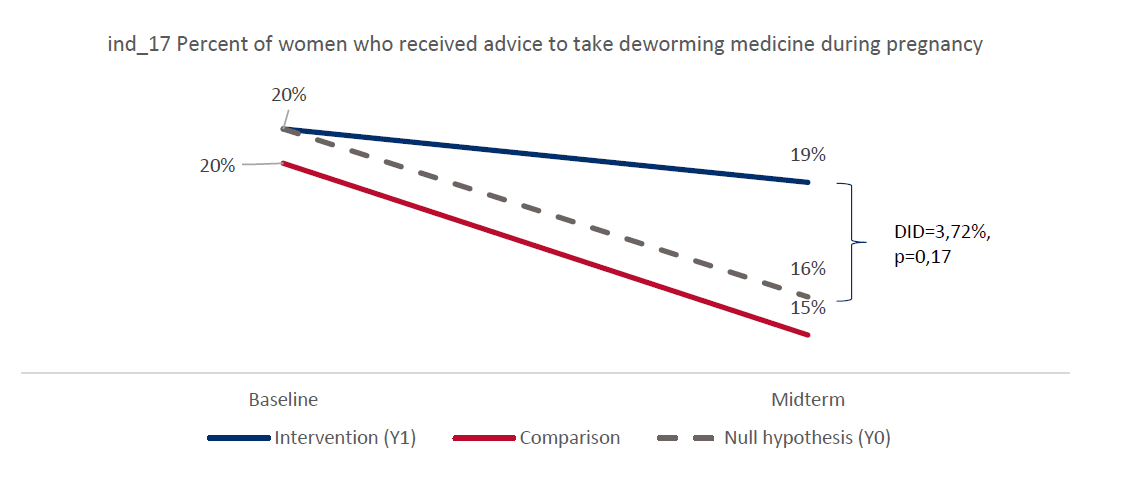
**

**
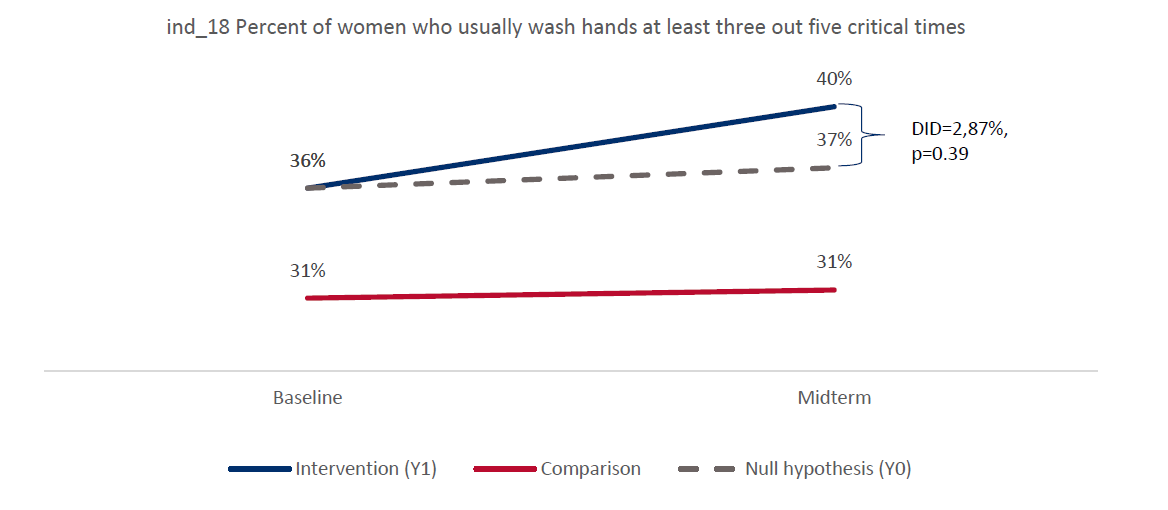
**

ind_19 Percent of households with soap and water at a handwashing station on premises

**
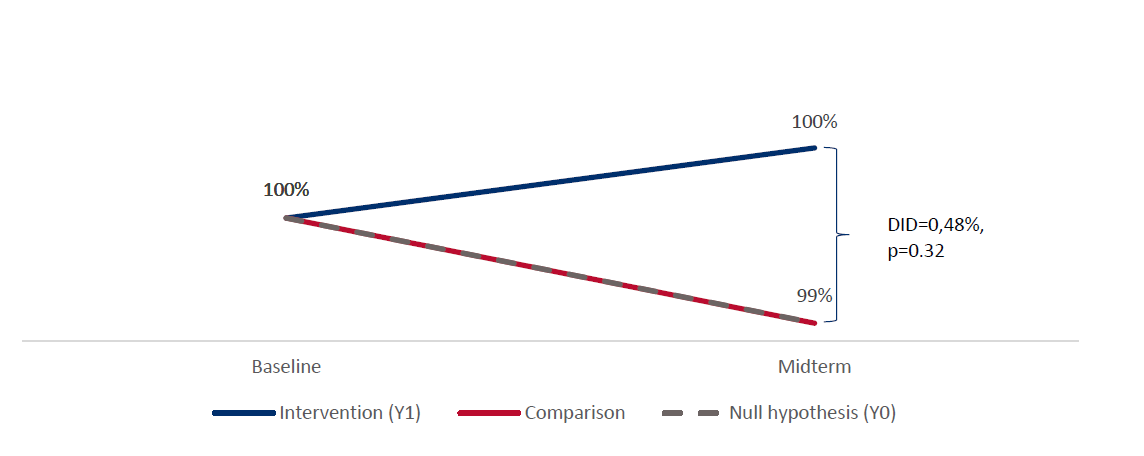
**

**
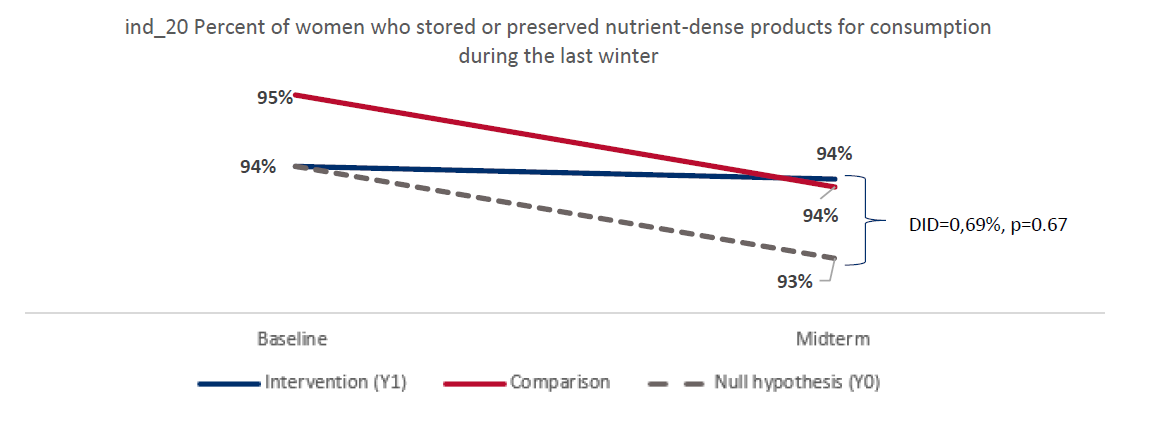
**

**Supplementary Material 5: Tables of Differences by Area**

**Table 5.1. Demographics**

| Characteristic | Baseline % | | Mid-term % | | Final % | |
| --- | --- | --- | --- | --- | --- | --- |
|  | Batken (n=1,241) | Jalal-Abad (n=849) | Batken (n=1,106) | Jalal-Abad (n=1,125) | Batken (n=966) | Jalal-Abad (n=958) |
| **Mother Age** | | | | | | |
| 18–24 | 27.6 | 22.7 | 22.6 | 21.6 | 23.2 | 23.3 |
| 25–29 | 34.8 | 35.3 | 34.5 | 36.7 | 35.5 | 33.7 |
| 30–39 | 34.7 | 38.3 | 40.0 | 39.3 | 39.1 | 38.4 |
| 40–49 | 2.8 | 3.7 | 2.9 | 2.4 | 2.2 | 4.6 |
| **Education** | | | | | | |
| None | 0.2 | 0.1 | 0.3 | 0.0 | 0.1 | 0 |
| Primary | 0.2 | 0.1 | 0.1 | 0.4 | 0 | 0.2 |
| Basic secondary (9 grades) | 3.8 | 8.5 | 6.2 | 12.0 | 7.5 | 9.5 |
| General secondary (11 grades) | 38 | 38.8 | 32.2 | 34.7 | 31.2 | 33 |
| Initial vocational education | 4.0 | 2.4 | 3.3 | 1.4 | 2.5 | 1.8 |
| Secondary specialized education | 25.5 | 22.4 | 26.5 | 25.1 | 25.1 | 26.9 |
| Higher education (incomplete or completed) | 28.3 | 27.8 | 31.5 | 29.4 | 33.8 | 28.6 |
| **Marriage/Partnership** | | | | | | |
| Married | 92.2 | 98.4 | 98.3 | 98.3 | 97.4 | 98.8 |
| Divorced | 0.6 | 0.7 | 0.9 | 0.9 | 1.7 | 0.7 |
| Single | 0.9 | 0.6 | 0.5 | 0.8 | 0.8 | 0.4 |
| Widow | 0.2 | 0.4 | 0.3 | 0 | 0.1 | 0.1 |
| **Child Age** | | | | | | |
| 0–5 months | 29.8 | 20.6 | 29.2 | 30.7 | 33.2 | 37.4 |
| 6–11 months | 24.3 | 29.2 | 26 | 28.3 | 33.9 | 37.6 |
| 12–17 months | 26.1 | 30.6 | 24.9 | 22.9 | 18.7 | 12.7 |
| 18–23 months | 19.8 | 19.6 | 19.9 | 18.1 | 14.2 | 12.3 |
| **Child Sex** | | | | | | |
| Male | 51.7 | 52.4 | 50.4 | 51.7 | 50.4 | 49.9 |
| Female | 48.4 | 47.6 | 49.6 | 48.3 | 49.6 | 50.1 |

**Table 5.2. Indicator changes in Batken**

| **Indicator** | **Baseline** | | | | **Midterm** | | | | **Difference (Intervention [M-B] – Comparison [M-B])*†** | **Final** | | | | **Difference (Full Intervention [F-M] – Light intervention [F-M])*** |
| --- | --- | --- | --- | --- | --- | --- | --- | --- | --- | --- | --- | --- | --- | --- |
|  | **Intervention** | | **Comparison** | | **Intervention** | | **Comparison** | |  | **Light Intervention** | | **Full Intervention** | |  |
|  | n | % | n | % | n | % | n | % |  | n | % | n | % |  |
| Percent of mothers of children <2 who took iron supplements for 90 days or more during their last pregnancy | 239 | 51.84 | 206 | 51.63 | 284 | 62.78 | 243 | 63.45 | -0.89 | 256 | 61.78 | 171 | 61.43 | -1.02 |
| Percent of mothers of children <2 who ate foods from 5 or more of 10 food groups in the previous 24 hours | 425 | 92.19 | 332 | 83.21 | 401 | 88.74 | 339 | 88.51 | -8.75* | 356 | 85.82 | 240 | 86.74 | 1.15 |
| Percent of children 6–23 months who ate foods from 5 or more of 8 food groups in the previous 24 hours | 313 | 63.62 | 263 | 69.39 | 254 | 65.30 | 269 | 68.27 | 2.8 | 213 | 56.76 | 157 | 58.15 | -1.59 |
| Percent of children 6–23 months receiving a minimum acceptable diet | 83 | 18.86 | 62 | 17.22 | 65 | 17.66 | 54 | 14.84 | 1.19 | 61 | 17.38 | 40 | 15.81 | 1.26 |
| Percent of children 6–23 months who ate iron-rich foods in the previous 24 hours | 278 | 56.50 | 241 | 63.59 | 255 | 65.55 | 259 | 65.74 | 6.9 | 207 | 55.17 | 159 | 58.89 | 3.53 |
| Percent of children 6–23 months who ate vitamin A-rich foods in the previous 24 hours | 295 | 59.96 | 255 | 67.28 | 250 | 64.27 | 247 | 62.7 | 8.9* | 209 | 55.70 | 160 | 59.26 | 5.13 |
| Percent of children 6–23 months who received food the minimum acceptable number of times for their age and breastfeeding status | 113 | 25.68 | 80 | 22.22 | 94 | 25.82 | 73 | 20.05 | 2.3 | 98 | 27.92 | 62 | 24.51 | 2.35 |
| Percent of children 0–23 months who were put to breast within one hour of birth | 470 | 62.92 | 326 | 66.26 | 442 | 73.39 | 376 | 75.35 | 1.38 | 401 | 68.48 | 273 | 72.37 | 1.92 |
| Prevalence of exclusive breastfeeding of children under six months of age | 129 | 50.39 | 60 | 52.63 | 129 | 59.45 | 45 | 41.67 | 20.02* | 119 | 56.13 | 65 | 60.36 | 22.01* |
| Percent of children 6–8 months who received semi-solid or solid food during the previous 24 hours (without sweet, processed products) | 76 | 87.36 | 55 | 84.62 | 78 | 86.81 | 49 | 77.78 | 6.29 | 87 | 81.31 | 56 | 82.35 | 10.08 |
| Percent of children 6–23 months who are still breastfeeding | 408 | 83.10 | 320 | 84.43 | 330 | 85.31 | 329 | 83.93 | 2.72 | 322 | 86.13 | 229 | 85.13 | 0.38 |
| Percent of children 0–5 months who consumed sugary or processed food in the previous 24 hours | 44 | 17.19 | 16 | 14.04 | 25 | 11.98 | 27 | 25 | -16.17* | 29 | 13.68 | 11 | 9.91 | -16.79* |
| Percent of children 6–23 months who consumed sugary or processed food in the previous 24 hours | 408 | 82.93 | 332 | 87.60 | 300 | 77.12 | 335 | 85.03 | -3.23 | 271 | 72.41 | 193 | 71.48 | -8.84* |
| Percent of children 0–5 months who consumed tea in the previous 24 hours | 32 | 12.50 | 18 | 15.79 | 20 | 9.68 | 19 | 17.59 | -4.63 | 20 | 9.43 | 11 | 9.91 | -7.44 |
| Percent of children 6–23 months who consumed tea in the previous 24 hours | 351 | 71.34 | 288 | 75.99 | 234 | 60.15 | 287 | 72.84 | -8.04* | 209 | 55.97 | 163 | 60.37 | -8.29 |
| Percent of women who received advice to take deworming medicine during pregnancy | 85 | 18.44 | 65 | 16.29 | 78 | 17.33 | 45 | 12.33 | 2.86 | 79 | 17.82 | 36 | 12.12 | -0.69 |
| Percent of women who usually wash hands at least three out five critical times | 189 | 41.00 | 123 | 30.83 | 197 | 43.81 | 132 | 36.07 | -2.43 | 147 | 32.74 | 101 | 34.01 | 9.00* |
| Percent of households with soap and water at a handwashing station on premises | 459 | 99.57 | 397 | 99.50 | 451 | 100 | 362 | 98.91 | 1.03 | 447 | 99.34 | 292 | 98.99 | 0.75 |
| Percent of women who stored or preserved nutrient-dense products for consumption during the last winter | 442 | 95.88 | 379 | 94.99 | 440 | 94.62 | 372 | 96.37 | -2.83 | 402 | 88.79 | 265 | 91.41 | 0.68 |
| Percent of women reporting increased decision-making power with husband and/or family | Not collected | | Not collected | | 129 | 95.56 | 112 | 98.25 | Not applicable (N/A) | 201 | 98.06 | 121 | 97.58 | -3.17 |

*Statistically significant at *p*<0.05.

**Table 5.2. Indicator changes in Jalal-Abad**

| **Indicator** | **Baseline** | | | | **Midterm** | | | | **Difference (Intervention [M-B] – Comparison [M-B])*†** | **Final** | | | | **Difference (Full Intervention [F-M] – Light intervention [F-M])*** |
| --- | --- | --- | --- | --- | --- | --- | --- | --- | --- | --- | --- | --- | --- | --- |
|  | **Intervention** | | **Comparison** | | **Intervention** | | **Comparison** | |  | **Light Intervention** | | **Full Intervention** | |  |
|  | n | % | n | % | n | % | n | % |  | n | % | n | % |  |
| Percent of mothers of children <2 who took iron supplements for 90 days or more during their last pregnancy | 155 | 42.94 | 165 | 41.09 | 201 | 52.34 | 240 | 51.28 | -0.79 | 166 | 55.70 | 227 | 50.67 | -3.97 |
| Percent of mothers of children <2 who ate foods from 5 or more of 10 food groups in the previous 24 hours | 319 | 88.37 | 357 | 88.61 | 336 | 87.50 | 409 | 87.58 | 0.17 | 255 | 85.86 | 380 | 84.82 | -1.12 |
| Percent of children 6–23 months who ate foods from 5 or more of 8 food groups in the previous 24 hours | 203 | 66.78 | 237 | 63.88 | 251 | 64.19 | 225 | 57.99 | 3.31 | 139 | 58.40 | 201 | 55.52 | 3.33 |
| Percent of children 6–23 months receiving a minimum acceptable diet | 46 | 15.97 | 53 | 15.36 | 71 | 19.94 | 52 | 14.77 | 4.56 | 33 | 14.80 | 74 | 22.84 | 13.21* |
| Percent of children 6–23 months who ate iron-rich foods in the previous 24 hours | 221 | 72.70 | 244 | 66.04 | 261 | 66.75 | 233 | 60.05 | 0.04 | 148 | 62.18 | 207 | 57.18 | 1.70 |
| Percent of children 6–23 months who ate vitamin A-rich foods in the previous 24 hours | 169 | 55.59 | 205 | 55.26 | 232 | 59.34 | 201 | 51.80 | 7.19 | 129 | 54.20 | 178 | 49.17 | 2.50 |
| Percent of children 6–23 months who received food the minimum acceptable number of times for their age and breastfeeding status | 64 | 22.22 | 83 | 24.06 | 92 | 25.84 | 74 | 20.96 | 6.72 | 57 | 25.56 | 105 | 32.41 | 11.73* |
| Percent of children 0–23 months who were put to breast within one hour of birth | 239 | 63.90 | 275 | 58.35 | 354 | 67.75 | 383 | 63.83 | -1.64 | 242 | 66.48 | 376 | 63.62 | 1.05 |
| Prevalence of exclusive breastfeeding of children under six months of age | 39 | 53.42 | 44 | 43.14 | 66 | 48.89 | 82 | 38.68 | -0.08 | 67 | 52.34 | 144 | 62.61 | 20.47* |
| Percent of children 6–8 months who received semi-solid or solid food during the previous 24 hours (without sweet, processed products) | 43 | 89.58 | 56 | 87.50 | 62 | 81.58 | 88 | 83.02 | -3.52 | 50 | 83.33 | 112 | 81.16 | -3.61 |
| Percent of children 6–23 months who are still breastfeeding | 236 | 78.41 | 306 | 82.48 | 319 | 82.01 | 315 | 81.19 | 4.89 | 199 | 84.32 | 313 | 86.70 | 3.20 |
| Percent of children 0–5 months who consumed sugary or processed food in the previous 24 hours | 5 | 6.85 | 15 | 14.71 | 15 | 11.11 | 30 | 14.15 | 4.82 | 10 | 7.81 | 21 | 9.13 | -1.72 |
| Percent of children 6–23 months who consumed sugary or processed food in the previous 24 hours | 270 | 88.82 | 316 | 85.44 | 311 | 79.54 | 286 | 73.71 | 2.46 | 167 | 70.17 | 230 | 63.54 | -0.80 |
| Percent of children 0-5 months who consumed tea in the previous 24 hours | 9 | 12.33 | 12 | 11.76 | 9 | 6.67 | 25 | 11.79 | -5.69 | 12 | 9.38 | 14 | 6.09 | -8.41* |
| Percent of children 6-23 months who consumed tea in the previous 24 hours | 233 | 76.64 | 257 | 69.27 | 268 | 68.54 | 245 | 63.14 | -1.97 | 143 | 60.08 | 175 | 48.34 | -6.34 |
| Percent of women who received advice to take deworming medicine during pregnancy | 83 | 22.99 | 90 | 22.28 | 87 | 22.19 | 76 | 16.67 | 4.81 | 62 | 21.83 | 84 | 18.63 | 2.32 |
| Percent of women who usually wash hands at least three out five critical times | 106 | 29.36 | 122 | 30.20 | 137 | 35.03 | 127 | 27.79 | 8.07* | 88 | 30.88 | 130 | 28.70 | 5.06 |
| Percent of households with soap and water at a handwashing station on premises | 359 | 99.45 | 401 | 99.50 | 391 | 99.49 | 455 | 99.56 | -0.01 | 284 | 99.65 | 450 | 99.34 | -0.38 |
| Percent of women who stored or preserved nutrient-dense products for consumption during the last winter | 332 | 91.97 | 382 | 94.80 | 381 | 93.86 | 409 | 91.91 | 4.78* | 234 | 90.00 | 401 | 89.91 | 1.65 |
| Percent of women reporting increased decision-making power with husband and/or family | Not Collected | | Not Collected | | 126 | 96.92 | 153 | 99.35 | N/A | 103 | 99.04 | 186 | 97.89 | -3.57 |

*Statistically significant at *p*<0.05.
